# Ras-dva small GTPases lost during evolution of amniotes regulate regeneration in anamniotes

**DOI:** 10.1038/s41598-018-30811-0

**Published:** 2018-08-29

**Authors:** Anastasiya S. Ivanova, Daria D. Korotkova, Galina V. Ermakova, Natalia Yu. Martynova, Andrey G. Zaraisky, Maria B. Tereshina

**Affiliations:** Laboratory of Molecular Bases of Embryogenesis, Shemyakin-Ovchinnikov Institute of Bioorganic Chemistry, Russian Academy of Sciences, Moscow, 117997 Russian Federation

## Abstract

In contrast to amniotes (reptiles, birds and mammals), anamniotes (fishes and amphibians) can effectively regenerate body appendages such as fins, limbs and tails. Why such a useful capability was progressively lost in amniotes remains unknown. As we have hypothesized recently, one of the reasons for this could be loss of some genes regulating the regeneration in evolution of amniotes. Here, we demonstrate the validity of this hypothesis by showing that genes of small GTPases *Ras-dva1* and *Ras-dva*2, that had been lost in a stepwise manner during evolution of amniotes and disappeared completely in placental mammals, are important for regeneration in anamniotes. Both *Ras-dva* genes are quickly activated in regenerative wound epithelium and blastema forming in the amputated adult *Danio rerio* fins and *Xenopus laevis* tadpoles’ tails and hindlimb buds. Down-regulation of any of two *Ras-dva* genes in fish and frog resulted in a retardation of regeneration accompanied by down-regulation of the regeneration marker genes. On the other hand, *Ras-dva* over-expression in tadpoles’ tails restores regeneration capacity during the refractory period when regeneration is blocked due to natural reasons. Thus our data on Ras-dva genes, which were eliminated in amniotes but play role in anamniotes regeneration regulation, satisfy our hypothesis.

## Introduction

Amniotes (reptiles, birds and mammals) differ from anamniotes (fishes and amphibians) in a reduced capacity to regenerate the body appendages. In fishes and amphibians the body appendages regenerate by epimorphosis, the essential steps of which are formation of specific wound epithelium (apical ectodermal cap) and the blastema beneath of it, i.e. the mass of dedifferentiated uni- or multipotent cells^1^. During last decades several signaling pathways, including Wnt, Notch, BMP, IGF and FGF, were shown to be involved in the regulation of regeneration in anamniotes^2–6^. Paradoxically, in spite of the fact that all these signaling pathways are very conservative among all vertebrates, the regeneration ability in amniotes is strongly reduced. It is thought that the reduction of regenerative capacities in amniotes is a result of some thin restructuring of their genetic network, without emergence or extinction of particular genes^7^. We have supposed that this could be also caused by the loss of some genes important for regeneration. We have shown recently that *Ag1*, the gene encoding a secreted protein from the family of Agr disulphidisomerase, which is crucial for regeneration of the body appendages and the forebrain development in anamniotes, was lost during evolution of amniotes^8,9^. An essential role for the regeneration of the newt limbs was also demonstrated for the close homolog of *Ag1*, *Agr*2^10^. These data confirm that besides rearrangement of the gene regulatory network, loss of genes, critical for regeneration, such as *Ag1*, could be also a reason for reduction of the regenerative capacities in amniotes.

To reveal possible novel regeneration regulators among genes lost during vertebrate evolution, we have tested now whether small GTPases Ras-dva1 and Ras-dva2, which are absent in all placental mammals and in many other amniotes, could be involved in the body appendages regeneration in fishes and amphibians. Our previous studies demonstrated that Ras-dva1 small GTPase is a functional partner of *Ag1* and *Agr*2^11,12^. Both *Agrs* and *Ras-dva1* participate in the regulatory feed-back loop, which is based on exchange of Fgf8 and Agr secreted proteins between anterior neural boarder cells (future telencephalon) and the adjacent cells of the preplacodal zone^13^. Within vertebrate subphylum, there are both *Ras-dva1* and *Ras-dva*2 in each species of fishes and amphibians (anurans as well as urodels). However, in all tested species of amniotes (reptiles, birds and lower mammals) only one *Ras-dva* gene (*Ras-dva1* or *Ras-dva2*) was found, and none of these genes were revealed in placental mammals.

Assuming these facts, we supposed that Ras-dva small GTPases may be involved in regulation of the regeneration. Indeed, we have now found out that these both genes are sharply activated upon the body appendages amputation in two model species: the adult *Danio rerio* and in the *Xenopus laevis* tadpoles, that indicates their involvement in the process of regeneration. Localization of *Ras-dva1* and *Ras-dva2* transcripts in the blastema cells suggests their involvement in the process of the blastema formation, which is crucial for regeneration success but lacks in amniotes. In support of the important role of *Ras-dva1* and *Ras-dva2* during regeneration, we have demonstrated that imitation of evolutionary loss of *Ras-dva1* and *Ras-dva2* in amniotes by down-regulation of these genes resulted in a significant retardation of the regeneration of *D. rerio* fins and *X. laevis* tadpoles’ tails. Consistently, inhibition of *Ras-dva1* and *Ras-dva2* functioning led to the inhibition of *Fgf20*, *Msx1b*, *Igf2b* and *Agrs*, expression of which is critical for the early steps of regeneration. In addition, we revealed obvious inhibition of mitotic activity in regenerating tail tissues under Ras-dva-down-regulation conditions. Intriguingly, over-expression of *Ras-dva* genes in non-regenerating *X. laevis* tadpoles was sufficient to restore regenerative capacity during the refractory period. These results confirm that in both fish and frog Ras-dva1 and Ras-dva2 small GTPases play important role in early steps of the body appendages regeneration. In turn, this supports our hypothesis that some genes lost during evolution of vertebrates could play role in regulation of regeneration in anamniotes and loss of them in the ancestors of amniotes in addition to rearrangement of the regeneration gene regulatory networks could have led to the reduction of regenerative capacities in extant amniotes.

## Results

### *Ras-dva* genes have disappeared in a stepwise manner during evolution of amniotes

**Figure 1:**
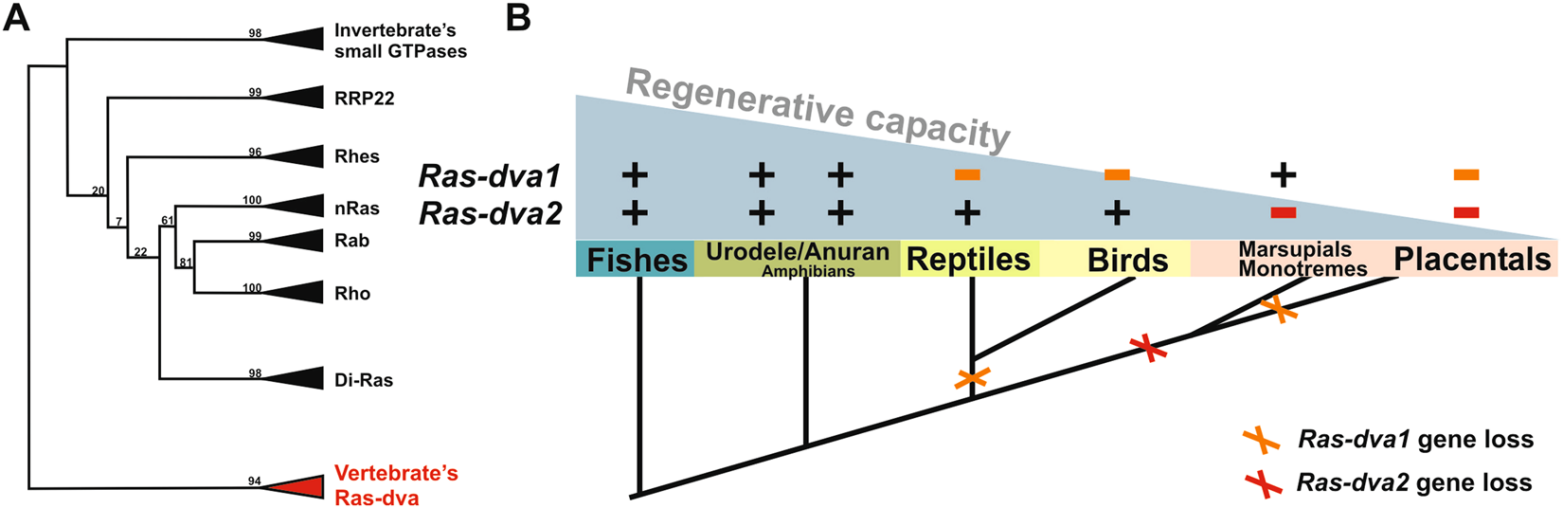
Genes of Ras-dva family of small GTPases were eliminated during vertebrates’ evolution. (**A**) Schematic version of the phylogenetic tree (full version see at Supplementary Fig. S1) of Ras-dva small GTPases and nearest groups of small GTPases, Ras, Rho, Rab *etc*. All vertebrate’s Ras-dva proteins form a separate bunch (bootstrap 94). The nearest homologs (41% of homology) are invertebrate small GTPases. (**B**) Scheme of *Ras-dva* genes elimination during vertebrates’ evolution. The presence of *Ras-dva1* or *Ras-dva2* genes in different classes is marked by “plus”, absence - by “minus”. The gray triangle demonstrates the tendency of impairment in regenerative ability during the evolution. Oblique cross indicates the loss of *Ras-dva1* or *Ras-dva2* gene. Notably, lack of *Ras-dva* genes correlates with the regenerative capacity decrease. Drawings were done by M.B.T.

We have demonstrated recently that Ras-dva small GTPases are represented in vertebrates by two sub-groups of proteins, Ras-dva1 and Ras-dva2^14^. Taking into account new data of genomic sequencing available in Gene Bank and Ensembl databases, we re-examined now the phylogeny of *Ras-dva* family members (for the phylogenetic tree see Supplementary Fig. S1, the scheme of it at Fig. 1A). As one may see, all Ras-dva1 and Ras-dva2 in vertebrates are clustered in the group well separated from Ras, Rho, Rab and other Ras-like small GTPases groups (Fig. 1A), which indicates some functional specificity of Ras-dva small GTPases. The scheme on Fig. 1B shows that both *Ras-dva1* and *Ras-dva2* are present in all classes of vertebrates from cyclostomes (lamprey) to amphibians including anurans, as well as urodeles. Interestingly, in reptiles, solely *Ras-dva2* was revealed, while only *Ras-dva1* was found in lower mammals, monotremes and marsupials. Placental mammals have no *Ras-dva* genes at all. Thus, this analysis revealed a clear tendency for a gradual loss of *Ras-dva* genes during the evolution of vertebrates, which correlates with a general tendency of decrease in regeneration capacity from cyclostomes to placental mammals (Fig. 1B). Basing on these data, we supposed that Ras-dva small GTPases could be involved in regulation of regeneration process.

### Studying of *Ras-dva1* and *Ras-dva2* temporal expression patterns during regeneration

**Figure 2:**
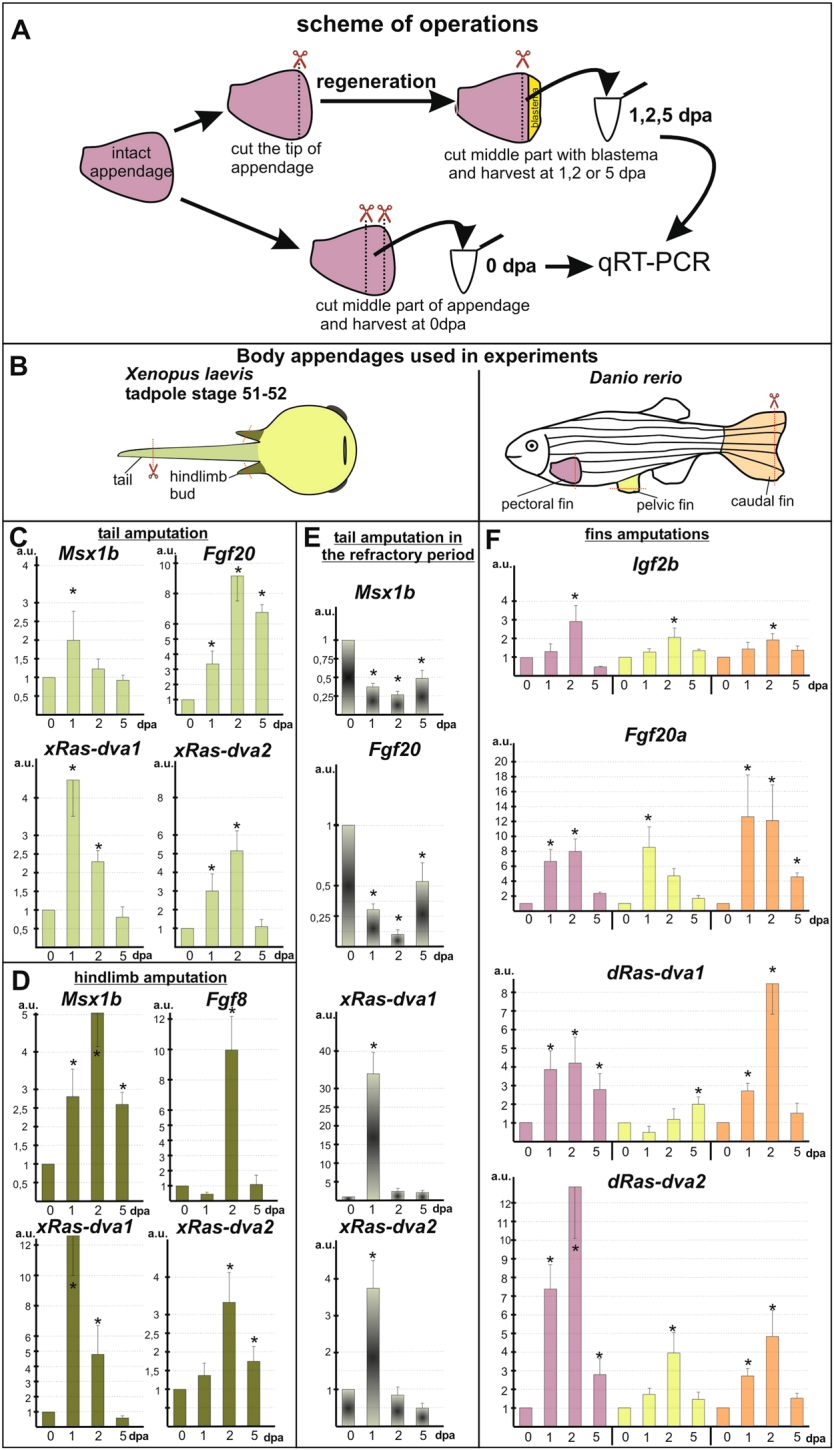
The qRT-PCR analysis of *Ras-dva1*, *Ras-dva2* and regeneration markers expression in *X. laevis* tadpoles and *D. rerio* adult fishes after the body appendages amputation. (**A**,**B**) Schemes of experiments. Drawings were done by M.B.T. (**C**,**D**) The results of qRT-PCR analysis of *xRas-dva1*, *xRas-dva2* and marker genes (*Msx1b, Fgf20* or *Fgf8*) expression dynamics during *X. laevis* tadpole (stage 51) tail (**C**) or hindlimb bud (**D**) regeneration at 0, 1, 2, 5 dpa (days post amputation). The geometric mean of expression of two reference housekeeping genes: *ornithine decarboxylase (ODC)* and *elongation factor 1alpa (EF-1alpha)* was used for normalization of the target genes expression levels. (**E**) The expression of *xRas-dva* genes and regeneration marker genes in tadpole tails amputated in the refractory period (stage 46) at 1, 2, 5 dpa in comparison to 0 dpa. (**F**) qRT-PCR analysis of *dRas-dva1, dRas-dva2* genes and *Fgf20a*, *Igf2b* markers expression pattern during the *D. rerio* fins regeneration on 1, 2 5 dpa in comparison to 0 dpa (the color of the fin on B correspond to the color of columns representing the gene expression in respective fin on **F**). All graphs represent means of quantification using total RNA derived from three independent samples. The value of normalized PCR signal in the 0 dpa sample, harvested immediately after amputation, was taken as an arbitrary unit (a.u.) in each series. Data are represented as mean ± SD, t-test, p < 0,05 (asterisk).

To investigate the involvement of *Ras-dva* genes in the body appendages regeneration, we analyzed by qRT-PCR their temporal expression patterns during regeneration in representatives of two classes of anamniotes, i.e. in the fish *Danio rerio* and the frog *Xenopus laevis*. In these model organisms, we analyzed expression of *Ras-dva* in the stump of the adult *D. rerio* fins and in the *X. laevis* tadpoles tails and hindlimb buds at 0, 1, 2 and 5 days post amputation (dpa). The pieces of stumps harvested just after amputation served as 0 dpa sample, considered as the basal controls with the level of gene expression characteristic to the non-amputated appendages (see Fig. 2A,B). The induction of regeneration in the amputated appendages was verified by the detection of transcripts of the up-regulated regeneration marker genes: *Fgf20a* and *Igf2b* in the *D. rerio* caudal fin^4,6^, *Msx1b* and *Fgf8* in the hindlimb bud and *Msx1b* and *Fgf20* in the tail of the *X. laevis* tadpoles^15^ at the stages of apical ectoderm cap and blastema formation, i.e. at 1 and 2 dpa.

As a result, we detected strong increase of *xRas-dva1* and *xRas-dva2* expression at 1 dpa in the *X. laevis* tail and hindlimb bud regenerates in comparison with the basal expression level at 0 dpa (Fig. 2C,D). Interestingly, at 2 dpa the expression level of *xRas-dva1* gene was decreased in contrast to that of *xRas-dva2*, which continued to be up-regulated. By 5 dpa, the expression of *xRas-dva2* was decreased and *xRas-dva1* gene expression returned to the basal level. In sum, the revealed temporal expression pattern of *Ras-dva* genes suggests their importance in the regeneration induction processes, particularly in the wound epithelium and blastema formation, which are implemented during 1 and 2 dpa.

*DRas-dva1* and *dRas-dva2* were also activated in 1 and 2 dpa stumps of the *D. rerio* fins, while their expression started to decrease (*dRas-dva1* in pectoral fin) or almost reached the basal level at 5 dpa in pectoral and caudal fins (Fig. 2F). Interestingly, in the pelvic fins *dRas-dva1* was not activated as strongly as in the pectoral and caudal fins. One may suppose that this could be due to the variations in the regeneration dynamics in different types of fins. Indeed, we found out that at the pelvic fin regenerated slower than the pectoral and caudal fins (see Supplementary Fig. S2). Noteworthy, whereas the expression level of *Ras-dva* genes in pelvic fins appeared to be only slightly activated or even decreased at 1 dpa, it was up-regulated at 2–5 dpa, which correlated well with the increase of the pelvic fin restoration rate at that time.

To further test the involvement of *Ras-dva* genes in regeneration, we analyzed the expression of *xRas-dva1* and *xRas-dva2* in stumps of the *X. laevis* tadpole’s tails, amputated during the so-called refractory period (at stages 45–47). During this period, the tail regeneration in *X. laevis* appears to be temporarily blocked due to natural reasons^3^. It is known that during the refractory period at 1 dpa only the wound healing takes place, but at 2 dpa the blastema formation signals are blocked^3,16,17^. We selected samples according to Msx1b and Fgf20 markers expression down-regulation and analyzed Ras-dva expression patterns. If the amputation of the tail was performed during this period, a sharp up-regulation of the *xRas-dva1/xRas-dva2* expression was observed in the stump only at 1 dpa, and it was followed by a sharp decline of the expression to the basal level (Fig. 2E, see Supplementary Fig. S3). Up-regulation of *xRas-dva1* gene at 1 dpa in refractory period tadpole was the highest among all time points analyzed. The observed induction of *xRas-dva1* and *xRas-dva2* genes on 1 dpa probably indicates participation of these genes in the wound healing pathways. At the same time, the following quick drop of the expression to the basal level (already at 2 dpa instead of normal 5 dpa), correlates well with the aforementioned inhibition of the blastema-inducing pathways at 2 dpa and thereafter the refractory period, which, in turn, confirms the possible involvement of *Ras-dva* genes in one of these pathways. Worth to mention that the same dynamics of *Ras-dva* genes expression was detected in hindlimbs after amputation at prometamophic stages (at stage > 57) (Fig. S3F), when regeneration is dramatically declined^15^.

In sum, the revealed temporal expression patterns of *Ras-dva* genes suggest the involvement of these genes in the early steps of regeneration in both tested anamniotic species.

### Studying of *Ras-dva1* and *Ras-dva2* spatial expression patterns during regeneration

**Figure 3:**
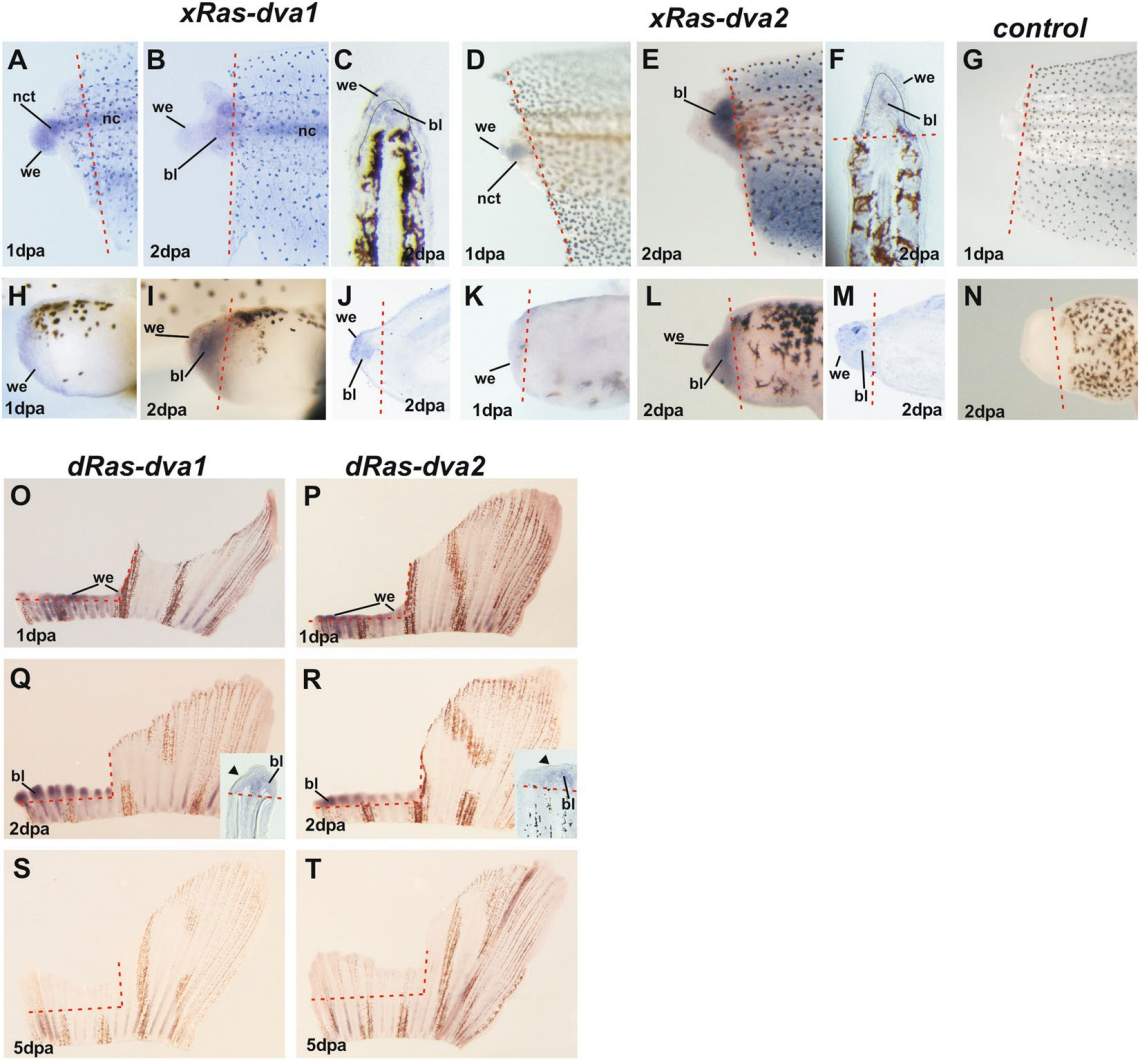
Analysis of *Ras-dva1* and *Ras-dva2* expression patterns during regeneration by whole-mount *in situ* hybridization. (**A**–**F**) Expression of *xRas-dva1* and *xRas-dva2* in *X. laevis* tadpole’s tails at 1 and 2 dpa in whole-mount tails (**A**,**B**,**D**,**E**) and on tail cryosections (**C**,**F**). At 1 dpa *xRas-dva1* transcripts were revealed in wound epithelium, notochord and notochord tip cells (**A**). At 2 dpa expression is shown in wound epithelium and blastema cells (**B,C**). Expression of *xRas-dva2* in *X. laevis* tadpole’s tails on 1 dpa is very poor in wound epithelium and notochord tip cells (**D**) and by 2 dpa it is strongly activated in blastema cells (**E,F**). (**G**,**N**) Tail 1 dpa and hindlimb 2 dpa stumps after whole-mount *in situ* hybridization with control (*sense xRas-dva1* + *sense xRas-dva2*) probe respectively. (**H**–**M**) Expression of *xRas-dva1* and *xRas-dva2* in *X. laevis* tadpole’s hindlimb stumps at 1 and 2 dpa respectively. At 1 dpa expression of both genes is seen in wound epithelium and by 2 dpa – in blastema cells as well (see cryosections **J**,**M**). Lateral view, distal to the left, dorsal to the top. (**O**–**T**) The *D. rerio* caudal fins with amputated left part after hybridization *in situ* with *dRas-dva1* (**O**,**Q,S**) or *dRas-dva2* (**P**,**R,T**) probe at 1, 2 and 5 dpa. The inserts on Q and R show ISH signal in 2 dpa fin cryosections. Black arrowhead – wound epithelium. Lateral view, distal to the top, dorsal to the left. Dashed red line marks the level of amputation, black dotted lines on C and F mark the wound epithelium boundary, we – wound epithelium, bl – blastema, nct – notochord tip.

To investigate spatial expression patterns of *Ras-dva1* and *Ras-dva*2 in tissues of the regenerating stump, we performed *in situ* hybridization experiments. To this end, we used the stumps of *D. rerio* caudal fins at 1, 2, 5 dpa or *X. laevis* tadpole tails and hindlimb buds at 1, 2 dpa and their cryosections. Consistently with the data of qRT-PCR analysis, activation of *Ras-dva1* and *Ras-dva2* expression was observed in all 1–2 dpa stumps (Fig. 3A–F, H–M,O–R). Noteworthy, now we detected *Ras-dva* genes expression in notochord in 0 dpa tail (Fig. 3S A,B 0 dpa), which was not seen in non-amputated whole-tadpoles samples previously probably due to bad accessiblity for *in situ* hybridization reagents.

As one may see on Fig. 3A, *xRas-dva1* is strongly expressed in the notochord, wound epithelium and notochord tip cells at 1 dpa. By 2 dpa, *xRas-dva1* expression is decreasing and transcripts are detected in the wound epithelium and blastema cells (Fig. 3B,C). The *xRas-dva2* transcripts are firstly detected at 1 dpa in notochord tip cells and in the wound epithelium (Fig. 3). By 2 dpa expression becomes stronger and is mainly localized in the blastema cells of tail regenerate (Fig. 3E,F). By 3 dpa no visible staining in tail is observed (not shown). If tail amputations were made in the refractory period *in situ* hybridization staining for *xRas-dva1* expression was strong in notochord tip and wound epithelium at 1 dpa, by 2 dpa expression was reduced and was close to basal expression in notochord. Expression of *xRas-dva2* gene in these samples was also activated by 1 dpa and decreased at 2 dpa (Fig. S3A,B).

Expression patterns of *Ras-dva* genes in *X. laevis* 1, 2 dpa hindlimb buds and *D. rerio* caudal fins (1, 2 dpa) are detected in the wound epithelium as well as in blastema area (Fig. 3H–M,O–R). By 5 dpa expression of *Ras-dva* genes is not detected in regenerating area of caudal fins or tails and hindlimbs (Fig. 3S,T and not shown).

**Figure 4:**
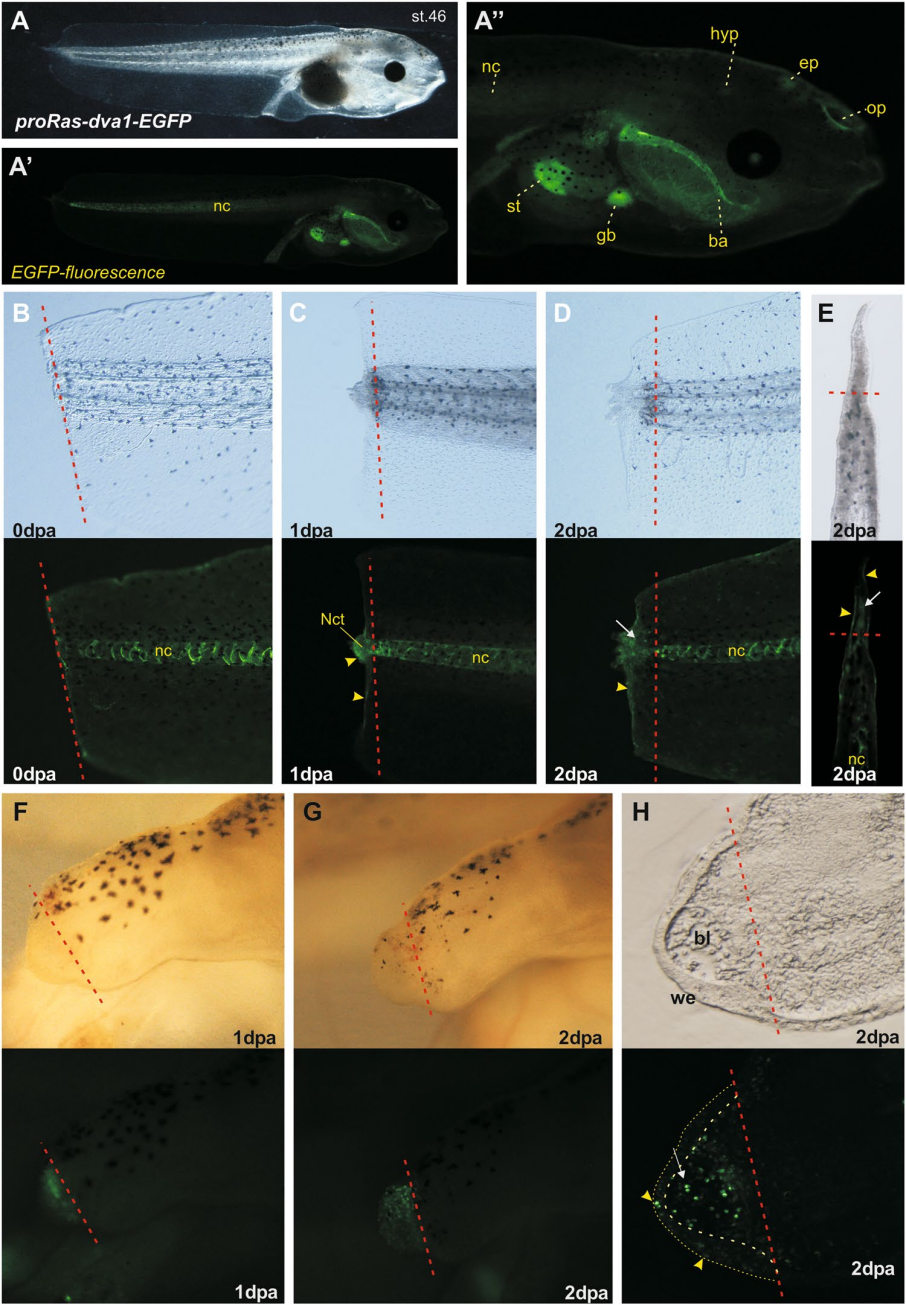
Promoter activation analysis in transgenic tadpoles, expressing *EGFP* under the *xRas-dva1* promoter control. (**A**,A’,A”) The transmitted light (**A**) and fluorescent (A’) images of whole transgene tadpole on stage 46 at 3 dpa, showing strong activity of *xRas-dva1* promoter in the notochord, gut and the head. The enlarged fluorescent image of head region (A”) demonstrates the *EGFP* fluorescence in ba - branchial arches, ep- epiphysis, gb - gall-bladder, hyp – hypophysis, nc – notochord, op - olfactory pits, st – stomach. (**B**–**E**) The transmitted light and fluorescent images of *proRas-dva1-EGFP* tadpole’s st.42 tail tip just after amputation 0 dpa (**B**), at 1 dpa (**C**), at 2 dpa (**D**) and its sagittal section (**E**). (**F**–**H**) The transmitted light and fluorescent images of proRas-dva1-EGFP tadpole st.52 hindlimb bud at 1 dpa (**F**), at 2 dpa (**G**) and its sagittal cryosection (**H**). Red dashed line indicates the amputation level. The yellow arrowheads point on cells of wound epithelium. The white arrows point on blastema cells. The yellow dashed lines on H indicate wound epithelium borders.

Unfortunately, despite detectable staining for *Ras-dva1/2* transcripts in the whole tadpole samples in wound epithelium and in the blastema cells, the *in situ* hybridization signal was weak in sections because of the overall low level of *Ras-dva* expression. Therefore, we used previously generated the transgenic line of frogs, which express *EGFP* under the control of *X. laevis Ras-dva-1* promoter fragment (4000 bp), to confirm the data of *in situ* hybridization with an alternative method. At the stage 46 the observed *EGFP* expression pattern in the transgenic tadpoles was in good agreement with the expression pattern of *xRas-dva1* gene, detected previously in the wild type *X. laevis* tadpoles by whole-mount *in situ* hybridization, including expression in olfactory pits, epiphysis (pineal gland), hypophysis (pituitary), branchial arches, stomach and gall-bladder^14^ (Fig. 4A,A’,A”). Also we detected EGFP expression in notochord consisnently with mentioned above *in situ* hybridization results. After amputation of the transgenic tadpoles’ tails at stage 42, we observed activation of *EGFP* fluorescence in the distal parts of the tail stumps already at 1 dpa, thus confirming the data of *in situ* hybrydization experiments (Fig. 4B,C). By 2 dpa, the *EGFP* signal was still strong (Fig. 4D) and localized in the wound epithelium and inside the regenerating tail. The fluorescent signals disappeared by 5–6 dpa (not shown). Importantly, sagittal vibratome sections of the 2 dpa tails of transgenic tadpoles revealed clear fluorescent signal in the wound epithelium and in blastema cells (Fig. 4E).

The hindlimb buds of transgenic tadpoles amputated at 51 stage also demonstrated activation of *xRas-dva1* promoter in distal parts of the regenerates at 1 and 2 dpa (Fig. 4F,G). The corresponding sagittal cryosections of the 2 dpa hindlimb bud revealed presence of fluorescent cells in the wound epithelium and in the blastema, thus also confirming the data of the whole-mount *in situ* hybridization experiments (Fig. 4H).

In sum, the obtained data show that *Ras-dva1* and *Ras-dva2* are activated in the regenerating body appendages of both fish and frog during 1–2 dpa in cells of the wound epithelium and blastema, which, in turn, indicates probable involvement of these genes in regulation of the regeneration.

If *proRas-dva1* tadpoles tail amputations were performed in refractory period high activation of *xRas-dva1* promoter was observed in wound epithelium and notochord tip at 1 dpa, but by 2 dpa only weak expression of *EGFP* was detected in the wound epithelium (see Fig. S3C–E). These data are consistent with *in situ* and qRT-PCR results on *xRas-dva1* dynamics after tail amputation in refractory period.

### Down-regulation of *Ras-dva1* or *Ras-dva2* in the amputated body appendages leads to suppression of regeneration

To determine whether activity of *Ras-dva1* and *Ras-dva2* is indeed necessary for the regeneration, we arranged a series of experiments on their down-regulation during the regeneration of *X. laevis* tadpole tail and *D. rerio* caudal fin. To inhibit translation of endogenous *X. laevis xRas-dva1* and *xRas-dva2* mRNAs, we injected the conventional morpholino anti-sense oligonuicleotides (MO) to these mRNAs mixed with the fluorescent tracer fluorescein dextran (FLD). The efficiency and specificity of *xRas-dva MOs* were preliminary tested and confirmed (Fig. 4S). To minimize effects of the MO in the anterior ectoderm, in which Ras-dva1 functioning is crucial for early development and consequently might influence the regeneration, we injected embryos in equatorial region, targeting MO predominantly into the cells precursor of the tail bud. Besides, we excluded from further statistical analysis the regenerates with significant developmental abnormalities, caused by injected *xRas-dva MO*. When the injected embryos at stages 40–41 (3 days post fertilization), the tails containing FLD tracer were amputated and effectiveness of their regeneration was analyzed by 8 dpa.

**Figure 5:**
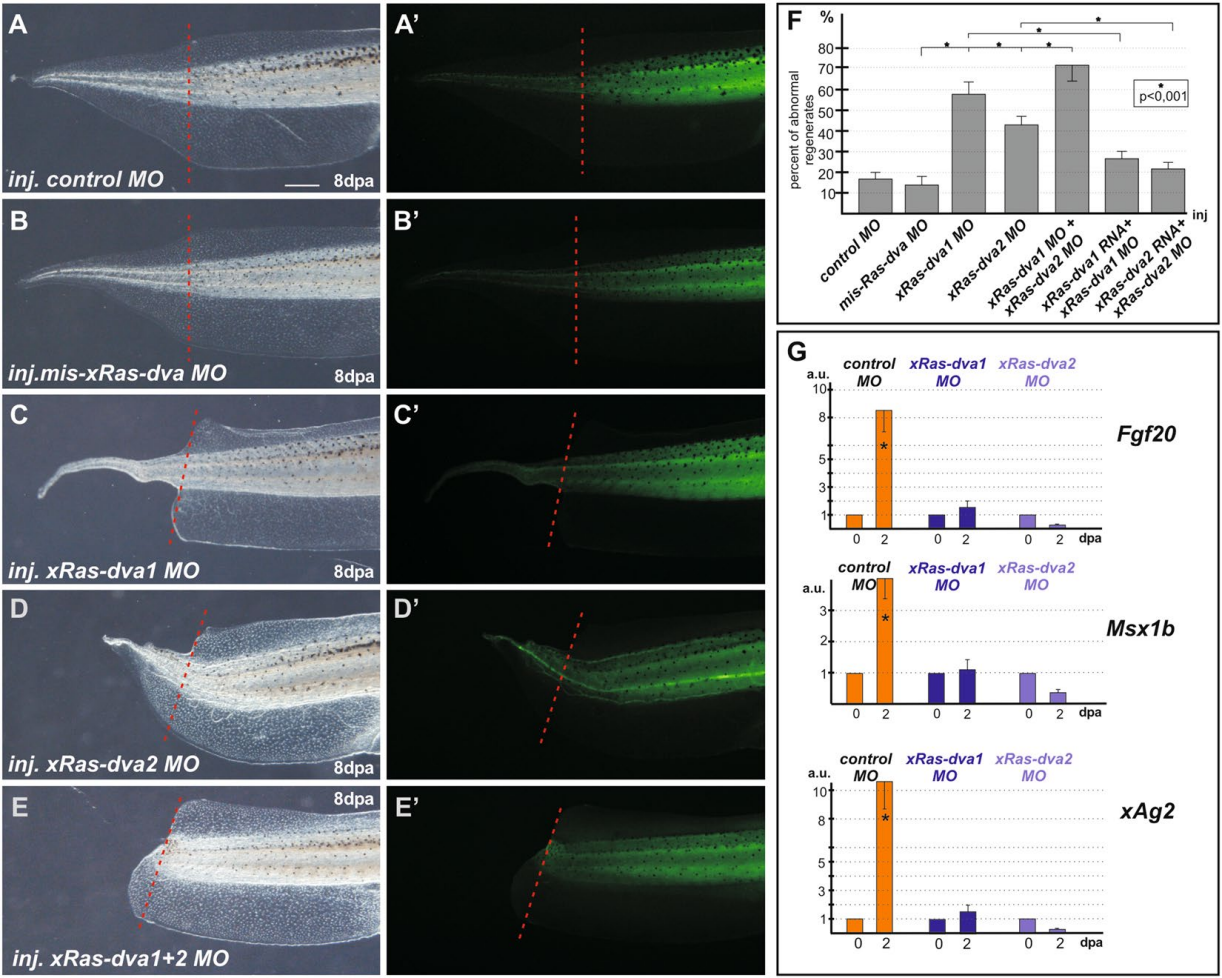
Down-regulation of *xRas-dva1* and *xRas-dva2* genes functioning results in abnormal regeneration of tadpole’s tails. (**A**–**E**) The transmitted light and fluorescent images of regenerated tails of 8 dpa tadpoles injected by solution of *control MO* (**A**), mismatched *mis-xRas-dva MO* (**B**), *xRas-dva1 MO* (**C**), *xRas-dva2* MO (**D**) or *xRas-dva1 MO* + *xRas-dva2 MO* (**E**) mixed with fluorescent tracer FLD. The red dashed line indicates the amputation level. Scale bar 0,5 mm. (**F**) Quantification of abnormal regenerates percentage of tadpoles, injected by different MO solutions or by the “rescue” mixtures of *xRas-dva1 MO* + *xRas-dva1 RNA*, *xRas-dva2 MO* + *xRas-dva2 RNA*. Error bars indicate SD. Statistical significance was determined with paired sample t-test, the results are statistically significant, p < 0,001 (asterisk). (**G**) qRT-PCR analysis of expression levels changes of regeneration markers *Fgf20, Msx1b* and also *Xenopus Ag1* homolog, *xAg2*, during the regeneration process (at 0 and 2 dpa) in amputated tails of tadpoles injected by *control*, *xRas-dva1* or *xRas-dva2 MO* solution. The value of normalized PCR signal in the 0 dpa sample, harvested immediately after amputation, was taken as an arbitrary unit (a.u.) in each series. Data are represented as mean ± SD, t-test, p < 0,05 (asterisk).

As a result, significant number of abnormally regenerating tails was detected in tadpoles injected with *xRas-dva1 MO* (58%) or *xRas-dva2 MO* (43%) (Fig. 5C,D,F). The highest percentage of abnormal regenerates (72%) was detected in tadpoles with down-regulation of both *xRas-dva1* and *xRas-dva2* genes (Fig. 5E,F). The following types of abnormalities were observed: (1) no dorsal or ventral, or both fins in the regenerated tail (Fig. 5C), (2) the retarded regeneration that caused significant tail malformations (Fig. 5D), (3) complete retardation of the regeneration (Fig. 5E). To simplify the statistical analysis these three types of abnormalities were summarized and considered during statistical analysis as a retarded regeneration (Fig. 5F). In contrast to the injections with *xRas-dva1* and *2* specific *MO*, only 17% and 14% of regenerates were abnormal in the groups of tadpoles injected with the generic *control* or *mis-matched xRas-dva MO* (the mixture of *mis- xRas-dva1* and *mis-xRas-dva2 MO*, which were unable to down-regulate x*Ras-dva* genes) respectively when inspected at 8 dpa (180–200 tadpoles of each type were analyzed in three independent experiments) (Fig. 5A,B,F).

The specificity of the effects of *xRas-dva1* and *xRas-dva2* MO injections was confirmed in “rescue” experiments, in which these MOs were co-injected with *xRas-dva1* or *xRas-dva2* mRNAs lacking the morpholino-target sites. As a result, in “rescue” groups the number of tadpoles with abnormal regeneration reduced two fold and was only 27% or 22% respectively out of 150 and 165 tadpoles analyzed in three independent experiments for each gene variant (Fig. 5F). These data confirm that normal functioning of *xRas-dva1* and *xRas-dva2* is necessary for the successful tail regeneration in *X. laevis* tadpoles.

As *Ras-dva* genes are active in embryos beginning from the late gastrula stage, one may suppose that the abnormal regeneration revealed at the tadpole developed from the *xRas-dva MO*-injected embryos could be the result of Ras-dva mis-functioning during much earlier stages. To confirm that the observed abnormalities were indeed the result of the MO-induced down-regulation of *Ras-dva* during regeneration, we used vivo-morpholino oligonucleotides (*vivoMO*) to *xRas-dva1* and *xRas-dva2*. Injections of *vivoMO* solutions were made directly into the distal part of the tails’ stumps immediately after amputation at tadpole’s stage 40–42. The results of testing of the *vivoMO* specificity and effectiveness can be found in Supplementary Information section (see Supplementary Fig. S4). Injections were performed once per day at 0, 1, 2 dpa, while statistical analysis of the regenerating tails was performed at 4 dpa (see Supplementary Fig. S5). As a result, we found out that among the tadpoles injected with *vivoMO* the statistical distribution of the malformed tail regenerates phenotypes were similar to those revealed in the tadpoles developed from embryos injected with the conventional MO (compare Fig. 5F and Supplementary Fig. S5). Obviously, all this confirms the validity of our knock-down experiments and suggest the involvement of Ras-dva small GTPases in regulation of the tail regeneration.

To characterize further the role of *xRas-dva1* and *xRas-dva2* during the tail regeneration, we compared the regeneration marker genes, *Fgf20* and *Msx1b*, expression in the tail stumps of tadpoles injected with the *control* or *xRas-dva1* and *xRas-dva2 MO*. In addition, we tested possible influence of *Ras-dva* down-regulation on the expression of *Agr* genes, which were recently shown to be regulated by Ras-dva1 during the early forebrain development and are strongly activated upon regeneration^9,13^.

Using qRT-PCR, we found out that the expression of *Fgf20*, *Msx1b* and *xAg2*, was suppressed in the stumps with down-regulated *Ras-dva1* and *2*. In contrast, in the tail stumps of tadpoles injected with the *control MO* strong activation of these genes was observed at 2 dpa (Fig. 5G). These results indicate that some Ras-dva-dependent processes can influence the expression of the early regeneration regulators, *Fgf20, Msx1b and Ag2*, and are involved at early steps of regeneration.

To examine the role of dRas-dva1 and 2 in regeneration of the body appendages in *Danio*, we inhibited translation of their mRNAs by injecting antisense *vivoMO* into the stumps of the amputated caudal fins. The efficiency and specificity of *dRas-dva vivoMOs* were preliminary tested and confirmed as described in the Supplementary Information (see Supplementary Fig. S6).

**Figure 6:**
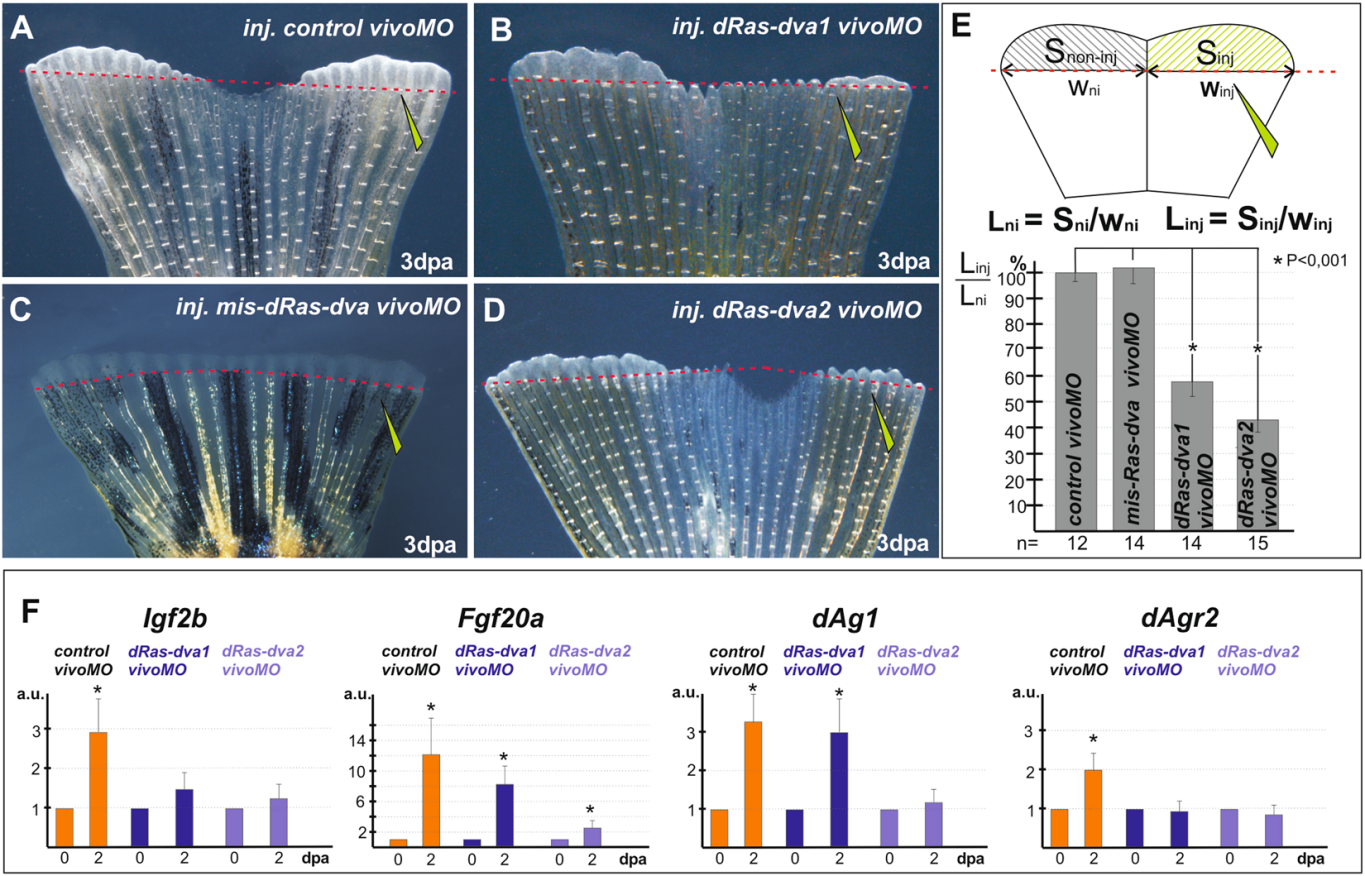
Down-regulation of *Ras-dva* genes by vivo-morpholino oligonucleotides inhibits the *D. rerio* fin regeneration. (**A**–**D**) Transmitted light images of regenerating *D. rerio* caudal fins injected in the right half by *control vivoMO* (**A**), *dRas-dva1 vivoMO* (**B**), *mis-dRas-dva1 vivoMO* (**C**) or *dRas-dva2 vivoMO* (**D**) at 3 dpa. Red dashed line indicates amputation level. Distal is upside, dorsal is to the left. Scale bar 200 µm. (**E**) Regeneration efficiency is calculated as normalized area of regenerated part of the caudal fin at 3 dpa injected by *vivoMO* divided by the normalized area of regenerated part that was not injected. Resulting values were taken as a percentage of the value obtained for control *vivoMO*. The scheme demonstrates how normalized length value <L> was calculated. S – square mean, W-width mean, ni- non-injected part, inj-injected part. Error bars indicate SD. Statistical significance was determined with two-tailed t-test, the results are statistically significant, p < 0,001 (asterisk). (**F**) qRT-PCR analysis of the expression of early regeneration marker genes *Igf2b* and *Fgf20a*, as well as *Agr* genes, *dAg1* and *dAgr2*, during the regeneration process (at 0 and 2 dpa) in the *D. rerio* caudal fins injected with *control*, *dRas-dva1* or *dRas-dva2 vivoMO*. The scheme of experiment is the same as described in Fig. 2A. The value of normalized PCR signal in the 0 dpa sample, harvested immediately after amputation, was taken as an arbitrary unit (a.u.) in each series. Dpa - days post amputation. Error bars indicate SD, t-test, p < 0.05 (asterisk).

As it is shown on Fig. 6, the fin injected with *dRas-dva1 vivoMO* or *dRas-dva2 vivoMO* in the right half demonstrated a significant retardation of regeneration at 3 dpa in the injected half of the fin in comparison to the non-injected half, where *dRas-dva* genes were not down-regulated (Fig. 6B,D). At the same time, caudal fins injected with *control vivoMO* or mismatched *mis-dRas-dva1 vivoMO and mis-dRas-dva2 vivoMO* into their right halves regenerated normally (Fig. 6A,C,E and not shown).

To check whether the retardation of regeneration observed in case of *dRas-dva1* or *dRas-dva2 vivoMO* injections could be caused by cell death, which was shown to be a nonspecific effect of some *vivoMOs*, we performed TUNEL assay to detect the number of apoptotic cells in the regenerating fins injected with *vivoMO*. As a result, no statistically significant difference in average concentration of apoptotic cells in regenerates was revealed between normally regenerating FLD- or *control vivoMO*-injected fins and their retarded parts that were injected with *dRas-dva1* or *dRas-dva2 vivoMO* (see Supplementary Fig. S6). Thus, we concluded that the observed effect of the retardation of the fin regeneration was not the result of cell death but rather the specific effect of inhibition of regeneration inducing signaling pathways in the absence of *dRas-dva* genes functioning.

In order to test if the dRas-dva functioning is required for the regeneration initiation in the fish at the molecular level, we analyzed the regeneration marker genes expression at 0 and 2 dpa in the caudal fin stumps injected by the *control* or *dRas-dva1* and *dRas-dva2 vivoMO* by qRT-PCR. As regeneration marker genes we used *Igf2b* and *Fgf20*, as well as *Agr* genes, *dAg1* and *dAgr2*, which were recently shown to be sharply induced at 1–2 dpa in *D. rerio* fins^8^. As a result, we found out that *Igf2b, Fgf20a* and *dAg1, dAgr2* expression were slightly or significantly suppressed at 2 dpa in the stumps of fins with the down-regulated *dRas-dva1 or dRas-dva2* respectively in comparison to the *control vivoMO* injected fin stumps (Fig. 6F).

In sum, one may conclude that in both fish and frog Ras-dva1/Ras-dva2 functioning during the first two days after the body appendages amputation are essential for maintenance of the correct signaling network governing the initiation of the regeneration.

### Down-regulation of *Ras-dva1* or *Ras-dva2* in the amputated body appendages inhibits cell proliferation but does not affect apoptosis

As we have shown, *Ras-dva* genes are essential for early steps of regeneration. To check what morphological abnormalities could appear upon *Ras-dva* knockdown in tadpoles’ tails at 1–2 dpa we made histological analysis of cryosectioned tails with normal and down-regulated *xRas-dva1* or xRas-dva2. As a result, we revealed that in 1 dpa tails of tadpoles injected by *xRas-dva1* or *2 MO* the wound epithelium was much thinner in comparison to control. By 2 dpa the amount of blastemal cells differed significantly in the control and in the Ras-dva-down-regulated tails (see Supplementary Fig. S7).

**Figure 7:**
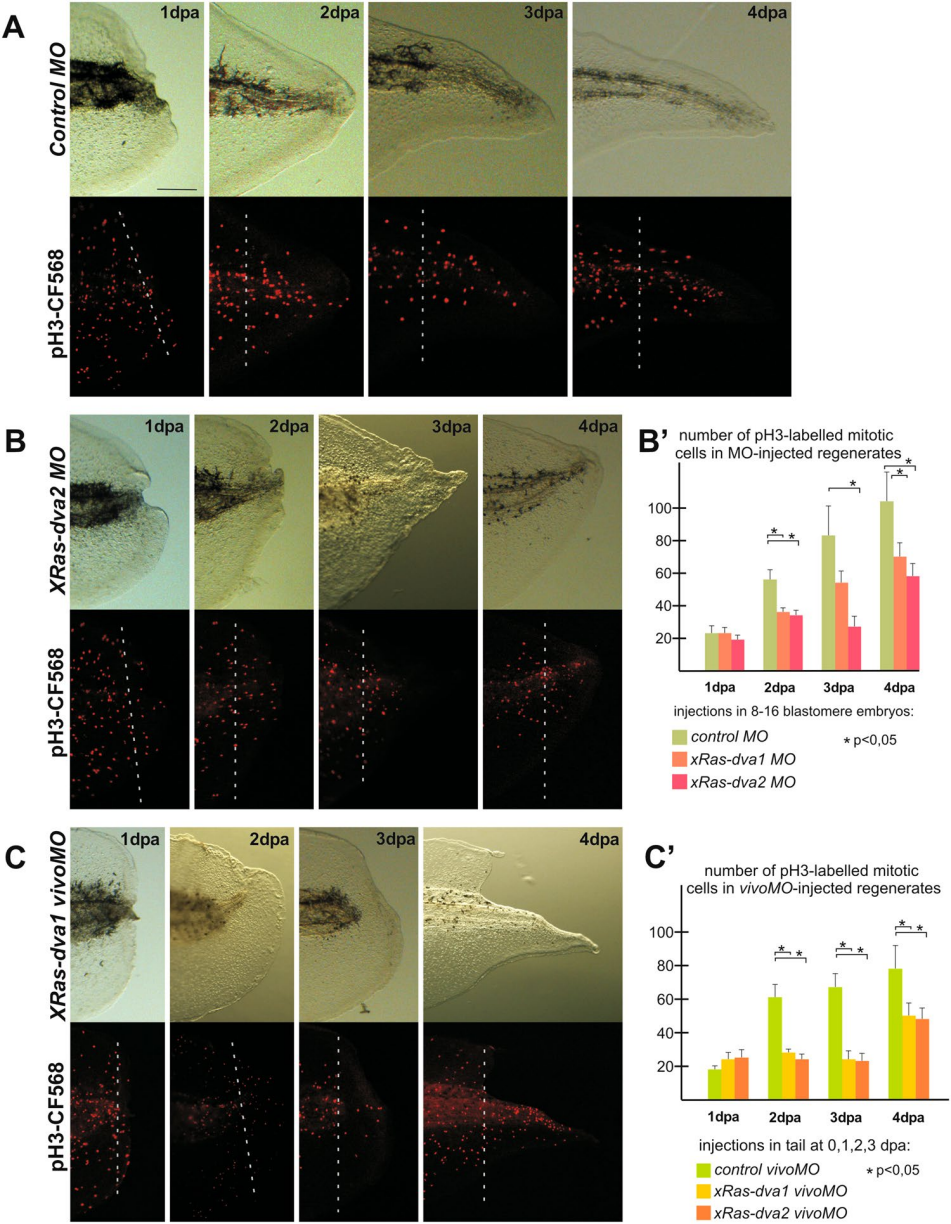
Analysis of mitotic cells patterns in amputated tails of tadpoles with normal and inhibited xRas-dva1 or 2 functioning during 1–4 dpa period. (**A**–**C**) Transmitted light and fluorescent images of distal areas of 1–4 dpa tails of tadpoles, developed from embryo injected by control MO (**A**) or x*Ras-dva2 MO* (**B**) or x*Ras-dva1 vivoMO* (**C**). (**A**) Immunostaining with primary rabbit anti-pH3 and secondary anti-rabbit antibodies conjugated with red fluorescent protein CF568 demonstrate gradual increase of mitotic activity in the regenerating area of tadpoles injected by *control MO* starting from 2 dpa (see B’ for statistics). (**B**) Transmitted light and fluorescent images of tadpoles injected by *xRas-dva2 MO* show inhibition of mitotic activity during 2–4 dpa. Scale bar 0,1 mm. (B’) Quantification of pH3-labbeled mitotic cells number in regenerates of 1–4 dpa tadpoles, developed from embryos injected by *control*, *xRas-dva1* or *xRas-dva2 MO*. Data of five independent experiments (5 tadpoles of each injection type were used in 1 experiment) were used for statistical analysis, statistical significance was determined by paired t test, p < 0,05 (asterisk). Error bars indicate SD. (**C**) Transmitted light and fluorescent images of tadpoles injected by *xRas-dva1 vivoMO* show strong inhibition of mitotic activity during 2–3 dpa. (C’) Quantification of mitotic cells number in 1–4 dpa regenerates of tadpoles injected at 0, 1,2 dpa *by control*, *xRas-dva1* or *xRas-dva2 vivoMO*. Data of five independent experiments (5 tadpoles of each injection type were used in 1 experiment) were used for statistical analysis, statistical significance was determined by paired t test, p < 0,05 (asterisk) Error bars indicate SD.

One may suppose that the observed abnormalities of regeneration in *X. laevis* tails with the down-regulated *Ras-dva* could be caused by the inhibition of cell proliferation. To verify this supposition, we analyzed mitotic cells profiles in the *X. laevis* tail stumps with normal or down-regulated *Ras-dva* functioning at 0–4 dpa in tadpoles injected with *xRas-dva1 MO*, *xRas-dva2 MO* or by *xRas-dva1 vivoMO/xRas-dva2 vivoMO*. Mitotic cells were detected by immunohistochemistry with antibodies to phospho-Histone H3 (Millipore), a marker of G2/M transition. At 1 dpa the mitotic profiles did not differ significantly in the control regenerates and in those of tadpoles injected with the conventional or *vivoMO*. However, at 2–3 dpa, during the blastema growth phase, the number of mitotic cells in regenerates with inhibited *Ras-dva1/2* was decreased in comparison with tails injected by the control MO (Fig. 7A–C,B’,C’). Noteworthy, injections of *xRas-dva1* or *2 vivoMO* at 0, 1, 2 dpa resulted in more strong mitosis inhibition in comparison to tadpoles developed from embryos injected with *xRas-dva1* or *2 MO* (Fig. 7B’,C’). This indicates more effective inhibition of *xRas-dva* by injections of *vivoMO* directly in the tail stumps at 0, 1, 2 dpa. At the same time, termination of *xRas-dva1/2 vivoMO* injections into tail stumps led to an increase of the proliferation in 4 dpa regenerates (Fig. 7C,C’). Importantly, the basic mitotic index (number of mitotic cells per area) in 0 dpa tails of tadpoles injected with the *control*, *xRas-dva1/2* conventional or *xRas-dva1/2 vivoMO* was approximately equal (not shown). This is one more confirmation of *MO* and *vivoMO* specificity and validation of the results of down-regulation experiments.

It was shown that programmed cell death is required during the first 24 h after amputation of tadpole’s tail for regeneration activation. Inhibition of caspase-dependent apoptosis results in a failure to induce proliferation in the growth zone, a mispatterning of axons in the regenerate and as a result abolished regeneration^18^. To check whether the decreased proliferation of cells in regenerates observed in case of down-regulation of *Ras-dva* genes could be caused by apoptosis inhibition, we performed TUNEL assay of the tail regenerates of the *X. laevis* tadpoles, developed from embryos injected with the *control* or x*Ras-dva1/2 MO* (see Supplementary Fig. S8). As a result, no statistically significant difference in average concentration of apoptotic cells in regenerates was revealed between all these samples. Thus, we concluded that the observed effect of the regeneration retardation was not the result of cell death inhibition, but rather was the specific effect of inhibition of some proliferation inducing signaling pathways in the absence of *xRas-dva* genes functioning.

### Over-expression of *Ras-dva* genes can restore the ability to regenerate tadpoles’ tails in the refractory period

**Figure 8:**
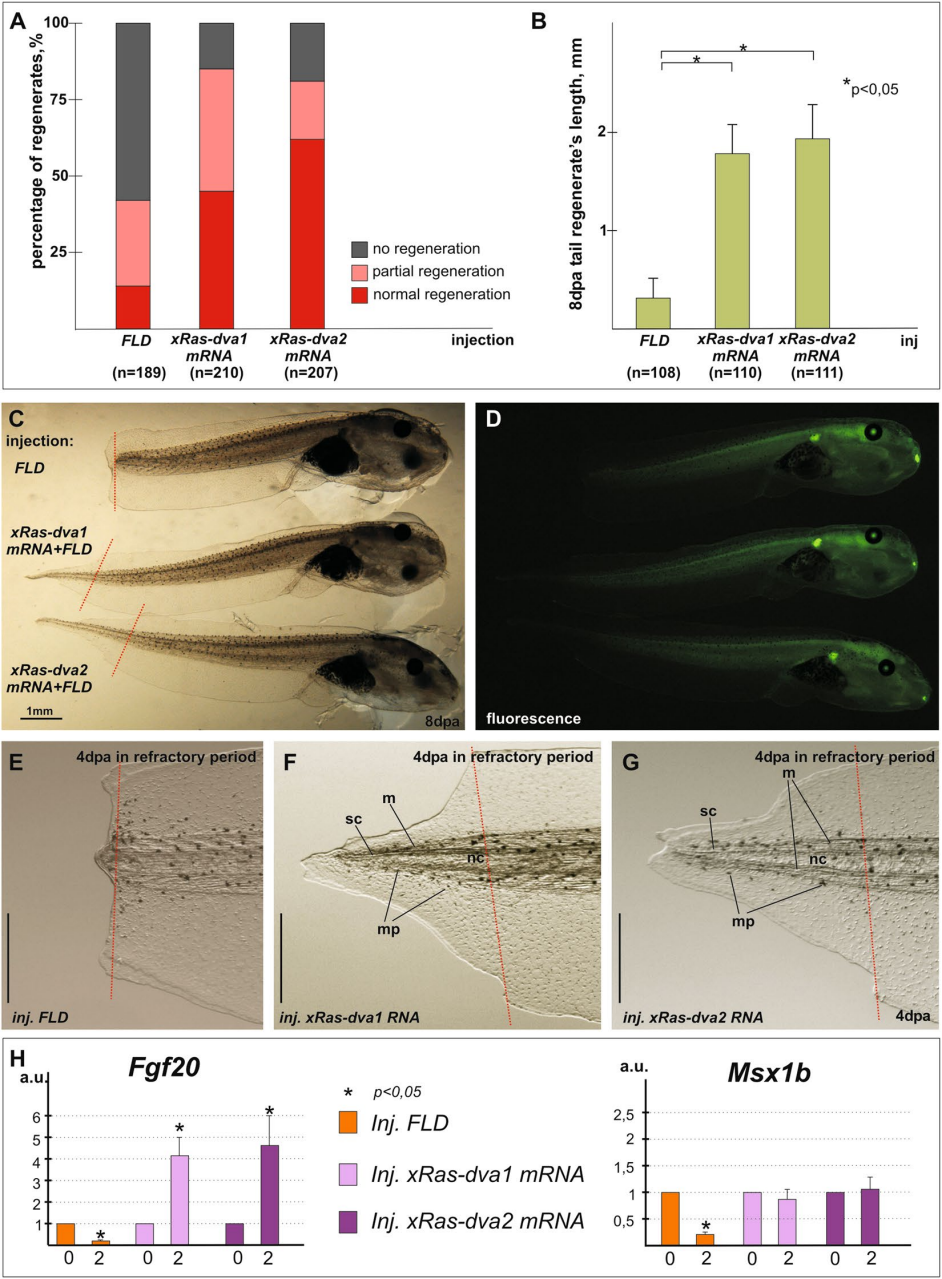
Rescue of the ability to regenerate tails during the refractory period in the *X. laevis* tadpoles. (**A**) Different rates of regeneration success in the refractory tadpoles with or without *Ras-dva1/2* overexpression. The average percent mean values of regenerating tadpoles with different success rates marked by colors: gray - no regeneration, pink – partial regeneration, red – normal regeneration, in tadpole butches injected with fluorescein (FLD, as control) or overexpressing *xRas-dva1* and *xRas-dva2 mRNAs*. n – number of injected tadpoles in three independent experiments. Differences of percent of normal regeneration between regenerates injected by FLD and mRNA are statistically significant, two-tailed t-test, p < 0,05. Percent differences of not regenerating tadpoles in FLD and mRNA injected tadpoles is statistically significant, two-tailed t-test, p < 0,05. (**B**) The measurements of the length of regenerated tail tips by 8 day after amputation of refractory tadpoles, developed from embryos injected by *FLD* (control) or *xRas-dva1* or *xRas-dva2 mRNA*. n – number of injected tadpoles in three independent experiments. Two-tailed t-test, p < 0,001 (asterisk). The transmitted light (**C**) and fluorescent (**D**) images of regenerated tails of 8 dpa refractory tadpoles injected with fluorescein (FLD) or *xRas-dva1 mRNA* + *FLD* and *xRas-dva2 mRNA* + *FLD*. FLD refractory tadpoles show regeneration arrest which is common for refractory period. Refractory tadpoles with *Ras-dva* genes overexpression effectively regenerate their tails. (**E**–**G**) The transmitted light images of regenerating tail tips of control tadpoles (FLD) or *xRas-dva1 mRNA* and *xRas-dva2 mRNA* at 4 dpa in refractory period show regeneration arrest in control tip but normal regeneration of notochord (nt), spinal cord (sc), muscles (m), melanophores (mp) in the tadpole’s tails with *xRas-dva1* or *xRas-dva2* overexpression. The red dashed line indicates the amputation level. Scale bar 1 mm. (**H**) qRT-PCR analysis of the expression of early regeneration marker genes *Fgf20* and *Msx1b* during the regeneration process (at 0 and 2 dpa) after amputation in refractory period in tadpoles tails injected with FLD or xRas-dva1 or xRas-dva2 mRNAs at early developmental stages. The scheme of samples harvesting is the same as described in Fig. 2A. The value of normalized PCR signal in the 0 dpa sample, harvested immediately after amputation, was taken as an arbitrary unit (a.u.) in each series. Dpa - days post amputation. Error bars indicate SD, t-test, p < 0.05 (asterisk).

Taking into account an important role of Ras-dva in the regeneration, we wondered whether the ectopic expression of these genes could rescue the regeneration capability of *X. laevis* tadpoles tail during the refractory period (st.45–47), i.e. when the regeneration is inhibited in normal development and the endogenous expression of *Ras-dva1* and *2* is strongly down-regulated at 2 dpa (Fig. 2E). To this end, we injected *mRNA* of *xRas-dva1* or *xRas-dva2* into 4–8 cells *X. laevis* embryos and then amputated tails of the tadpoles developed from these embryos at stage 46. The presence of ectopic *Ras-dva1* or *2 mRNA* in refractory tadpole’s tails was confirmed by *in situ* hybridization (see Supplementary Fig. S9). In addition, to control the over-expression of *xRas-dva1 in vivo*, we injected mRNA encoding for the fusion of *xRas-dva1* with *EGFP*^19^, which allowed us to trace the expression of the injected *mRNA* before and after amputation (not shown and see Supplementary Fig. S9). The translational activity of exogenous mRNA in refractory tails was tested using flag-tagged *xRas-dva1* and *2* mRNAs and Western blotting (see Supplementary Information and Fig. S10).The efficiency of the tail regeneration was examined at 3–4 dpa and classified into 3 grades: blocked - no regeneration, partial – the regenerates were asymmetric, shortened or curved, normal - normal regeneration. As a result, we observed that in the control group of tadpoles injected only with FLD water solution, the percentage of the blocked tail regeneration was notably higher then in tadpoles with over-expression of *xRas-dva1* or *2*. At the same time, in *xRas-dva*-over-expressed groups the percentage of tadpoles with normal regeneration was significantly increased, up to 45–62%, in contrast to 14% of normal regeneration in the control group (Fig. 8A). The percentage of partial regenerates did not change significantly. Measurements of the tail regenerating bud length in control and *Ras-dva* over-expressed tadpoles at 4 dpa revealed significant differences (Fig. 8B–D). The analysis of regeneration of diverse tail tissues at 4 dpa showed that *xRas-dva* over-expression restored the regeneration of all tail tissues, including notochord, neural tube, dorsal and ventral fins, muscles and melanophores in refractory tadpoles (Fig. 8E–G). The qRT-PCR analysis of the expression of early regeneration marker genes *Fgf20* and *Msx1b* during the regeneration process (at 0 and 2 dpa) after amputation in refractory period in tadpoles tails injected with FLD or *xRas-dva1* or *xRas-dva2 mRNAs* at early developmental stages have shown that *Ras-dva* genes can partially restore *Fgf20* expression activation and prevent down-regulation of *Msx1b* expression (Fig. 8H). These data demonstrate that under Ras-dva-overexpression in refractory period normal regeneration molecular processes can be detected. It is likely that regulatory activity of Ras-dva small GTPases (if their expression is not inhibitied) is sufficient to maintain tail regeneration ability in refractory period tadpoles.

## Discussion

We present here several lines of evidence that small GTPases Ras-dva1 and Ras-dva2, that were eliminated in a stepwise manner during evolution of amniotes, play important role in regulation of regeneration of the major body appendages in fish and frog.

In sum, the phylogenetic analysis indicates that the presence in a given species of both *Ras-dva1* and *Ras-dva2* correlates well with a good regenerative capacity in this species, while the loss of even one of *Ras-dva* genes coincides with the decreased regeneration capacity (Fig. 1B). Importantly, our experiments have shown that down-regulation of any one of two *Ras-dva* genes, *Ras-dva1* or *Ras-dva2*, is sufficient to inhibit regeneration. This is consistent with the fact that the absence of a single *Ras-dva* gene, i.e. *Ras-dva1*, in reptiles and birds or *Ras-dva2* in marsupials/monotremes, correlates with the decline of a robust regeneration capacity in these species^20,21^.

The detected *Ras-dva* expression patterns as well as results of the gain- and loss- of- function experiments indicate the involvement of these genes in regulation of the early steps of regeneration.

First, quick increase of the expression of both genes was detected at wound epithelium (1 dpa) and blastema formation steps (2 dpa) of the fish fins, the frog tadpole tails and hindlimb buds by three methods, including qRT-PCR, whole-mount *in situ* hybridization and tracing of EGFP driven by *xRas-dva1* promoter in transgenic tadpoles are consistent with the above statement. As one may see *Fgf8*, *Fgf20*, *Msx1* or *Igf2b* expression levels are also temporary up-regulated and they differ from each other as their functioning is necessary at different moments of regeneration induction. Noteworthy that *Ras-dva1* expression upregulation pattern in *Xenopus* tail correlate well with that of *Msx1b* and *Ras-dva2* – with that of *Fgf20*. As it was shown, Msx1b regulates cell proliferation of notochord and spinal cord and its function is necessary at 1–2 dpa. Down-stream of BMP signaling acts Fgf, which also controls cell proliferation during regeneration blastema formation^5^. In *Danio rerio* fins *Ras-dva* genes are up-regulated mostly by 2 dpa correlating with *Igf2b* and *Fgf20a* factors peaks, pointing on probable functional necessity exactly at blastema formation stage. In *Xenopus laevis* refractory period regeneration blockage is marked by strong down-regulation of regulators involved in the main regeneration step – blastema formation. We showed that during this period *Ras-dva* genes are activated at the wound epithelium formation stage (1 dpa), but sharply down-regulated at the stage of blastema formation (2 dpa), pointing on their involvement in blastema induction at regeneration-positive stages. As genes of Ras-dva were eliminated in evolution one may suppose that the most important was the loss of their blastema-inducing activity. The lack of this activity together with some other inhibitory pathways (like those suppressing blastema during the refractory stage), could lead to the efficient suppression of blastema formation and, as a result, the reduction of the regeneration capacity.

Second, a retardation of regeneration of all the tested body appendages in fish and frog was observed when either *Ras-dva1*, or *Ras-dva2*, or both genes were down-regulated by injections of the anti-sense morpholino oligonucleotides. The same regeneration malformations point on absence of Ras-dva functional redundancy. Meaning that primary structure of Ras-dva small GTPases is 82% homologous, it is unlikely that signaling downstream of Ras-dva1 and Ras-dva2 is different. This is puzzling but if we recall expression patterns of these genes we can see that *xRas-dva1* expression level in regenerating tail is highest at 1 dpa while *xRas-dva2* – is peaked at 2 dpa. This may explain that down-regulation of *xRas-dva2* can not be rescued by insufficient level of expressing *xRas-dva1* and results in regeneration impairment and vice versa. At the same time over-expression of either *xRas-dva1* or *xRas-dva2* can rescue regeneration refractory blockage, pointing on possibility of down-stream signaling commonality. The regeneration retardation was accompanied by reduction of expression of such important regulators of regeneration as *Fgf20*, *Igf2b*, *Msx1b* and *Agrs*. This indicates that Ras-dva1/2 function up-stream of them and are involved in regulation of processes controlled by these genes, in particular in the formation of the wound epithelium and blastema, which are crucial for the successful regeneration^3,4,6,8,9^. Consisnently with *Msx1b* down-regulation under xRas-dva knock-down we observed retardation of notochord and spinal cord regrowth and also abnormally thin wound epithelium in contrast to normal regeneration at 1 dpa tails. At 2 dpa Ras-dva-induced Fgf20 inhibition resulted in lower blastema cells dencity and low level of tail re-growth. Noteworthy, down-regulation of *Ras-dva2* led to a more significant inhibition of the regeneration marker genes at 2 dpa. This indicates that *Ras-dva2* functioning is important for maintenance of these genes’ expression at stage of the blastema formation (2 dpa). Besides, we demonstrated that during the blastema growth phase (at 2–3 dpa), Ras-dva functioning is necessary for proper induction of mitosis. Consistently, we revealed that Ras-dva small GTPases regulate proliferation in the blastema growth zone. In addition, we detected the thinning of the wound epithelium at 1 dpa and a reduced number of blastemal cells at 2 dpa and also the decline in regeneration of the *X. laevis* tail fins in the case of *Ras-dva* down-regulation. In sum, imitation of amniotic lack of *Ras-dva* genes by loss-of-function experiments in anamniotes demonstrates the dependence of the regenerative capability from normal functioning of *Ras-dva* genes.

Finally, over-expression of *xRas-dva* genes appeared to be able to rescue *X. laevis* tadpole’s tail regeneration during the refractory period, when the regeneration is normally blocked. One may suppose that over-expression of *Ras-dva* genes could disturb developmental timing and that *Ras-dva* over-expressing tadpoles at stages 45–47 may actually represent st. 42 or st. 51 in controls, where regeneration is normal. But it is known that refractory period stages 45–47 are characterized by a determined set of developmental parameters, in particular the intestine spiralization, showing 1.5 revolutions. We determined the refractory period beginning corresponding to these developmental characteristics in all groups of injected by FLD or RNA embryos. We didn’t detect that *Ras-dva* overexpression caused any shift in development timing of the whole embryo. The effect of regeneration ability maintenance in refractory period under *Ras-dva* overexpression is most likely obtained due to specific Ras-dva-induced shift in regulation of signaling networks making them active as at 42 or 51 stage. In support of this we detected partial up-regulation of *Fgf20* and *Msx1* maintenance of expression levels in *Ras-dva* over-expressing tadpoles in contrast to control not-regenerating tadpoles with down-regulated *Fgf20* expression (Fig. 7H). So, Ras-dva functioning is necessary and sufficient for regeneration induction in case of presence of all signaling network players essential for regeneration.

It is known that urodele amphibians are characterized by extremely strong regeneration capacity^22^. This feature makes urodeles a popular model for studding the mechanisms of the regeneration. In particular, an important role of *Agr2*, whose expression has been shown now to be regulated by Ras-dva in *X. laevis* and *D. rerio*, was demonstrated for the successful forelimbs regeneration in the adult newt and axolotl^10^. Recently, Bryant and colleges assembled and annotated a *de novo* transcriptome using RNA-sequencing profiles for a broad spectrum of *Ambystoma mexicanum* tissues, in particular of distal and proximal forelimb blastemas^23^. Consistently, *Agrs* and *Ras-dva* transcripts were identified in the distal and proximal blastema transcriptome samples of the axolotl regenerating forelimb (https://www.ncbi.nlm.nih.gov/bioproject/PRJNA300706). Obviously, this fact indirectly confirms participation of the *Ras-dva* genes network in the early blastema organization process in urodeles. However, further functional analysis of these genes during urodeles regeneration would be important.

The expression dynamics and physiological role of *Ras-dva* genes during regeneration resemble those of *Ag1* and *Agr2*. In our previous studies, we revealed a tight correlation of *Ag1*, *Agr2* and *Ras-dva1* expression and functioning during early development of the forebrain in the *X. laevis* embryo^13^. Besides, we have shown that during *Xenopus* tail and hindlimb regeneration *Ag1* gene is also up-regulated at 1–2 dpa and its transcripts are localized in wound epithelium and blastema cells^9^. Accordingly, one may suppose that a tight functional linkage between these genes may result in their dysfunction if one of them would disappear in evolution. In turn, if such a dysfunction appears to be useful for an organism for some reasons and is picked up by the positive selection, it may allow further stepwise elimination of other tightly linked genes. Indeed, as the activities of these genes would be no longer necessary to maintain its functionality, these genes, if not participating in other processes, will be degraded due to the accumulation of spontaneous mutations and finally could disappear completely during evolution.

An example of this scenario is probably observed in case of *Agr* and *Ras-dva*, when extinction of *Ag1* in amniotes correlates with elimination of one of *Ras-dva* genes in reptiles, birds and lower mammals and both *Ras-dva1* and *Ras-dva2* in placental mammals. Our data revealed that the reduction in the body appendages regenerative abilities may be caused by down-regulation of *Ras-dva* genes in fish or frog. This allows one to suppose that disappearance of *Ag1* accompanied by a stepwise loss of *Ras-dva* and probably some other genes helped to canalize the process of diminishing of regenerative capabilities in amniotes together with rearrangement of the regeneration gene regulatory networks.

An important question may arise in connection with the revealed phenomena of *Ag1* and Ras*-dva* disappearance during vertebrates’ evolution, namely, what could be the benefit that was able to compensate such an obvious drawback as a decrease in regenerative capacity? We suppose that at least one of such benefits could be related to the progressive development of the forebrain in amniotes^24^. Indeed, as we demonstrated earlier, *Ag1* and *Ras-dva1* are involved in the forebrain development in anamniotes^11,13^. Assuming this fact, one may suppose that elimination of these genes in evolution of amniotes might lead to the removal of some molecular mechanisms that prevent further progressive development of the forebrain. In that case, loss of *Ag1* and *Ras-dva* in amniotes could be considered as a peculiar payment for the progressive evolution of the forebrain in expense of the reduction of the regenerative capabilities.

## Materials and Methods

### Manipulations with tadpoles and fishes

Animal experiments were performed in accordance with guidelines approved by the Shemyakin-Ovchinnikov Institute of Bioorganic Chemistry (Moscow, Russia) Animal Committee and handled in accordance with the Animals (Scientific Procedures) Act 1986 and Helsinki Declaration. *X. laevis* tadpoles and *D. rerio* fins were obtained, amputated and harvested as we described previously^8,9^. The amputation of *Xenopus* tails or hindlimb buds were performed using Vannas microscissors and amputation of *Danio rerio* fins were made by the razor blade.

### Phylogenetic analysis

Ras-dva homologs in different species were identified as a result of the homology search of available genetic databases with the on-line Blast and Ensemble programs by accounting for specific motifs of Ras-dva protein sequences^11^. *Xenopus tropicalis* and *D. rerio* Ras-dva1 and Ras-dva2 protein sequences were used as queries in most cases. The orthology between *Ras-dva1* and *Ras-dva2* in different species was established through homology analysis of proteins and investigation of local genomic syntheny for each gene. The *Ras-dva1* synthenic group consisted from the following genes: *telo2, spsb3, eme2, ern2, asb8, rab11* and *tip3*. The synthetic group of *Ras-dva2* gene consisted from genes S*ept9, tnrc6c, Sec. 14L1, mfsd11, ube20, rhbdf2, foxj1*. For phylogenetic tree construction see Supplementary Materials and Methods and Supplementary Fig. S1.

### qRT-PCR, *in situ* hybridization

Samples preparation for *in situ* hybridization, qRT-PCR and qRT-PCR protocol were described in detail previously^8,9^. For sequences of primers used in qRT-PCR see Supplementary Information. The *in situ* hybridization was performed as described in^25^. As the notochord is not so easily accessible for *in situ* hybridization reagents the expression in notochord was not detected by us earlier. Interestingly, *in situ* hybridization at 0 dpa, just after amputation (when the notochord was open for reagents) resulted in good notochord staining for *xRas-dva1* or *xRas-dva2* expression (see Fig. S3A and B 0 dpa), but at 1 dpa, when wound epithelium was formed and accessibility of notochord for reagents was reduced and the BMP signal was not strong in notochord. If we used more prolonged proteinase K 15 minutes) and each PBS 0,2% Tween (10 minutes) and staining treatments (BMP + levamizol 24 hours) we were able to detect expression of *Ras-dva1/2* in notochord at 1 and 2 dpa also.

### *XRas-dva1* promoter cloning, transgenic tadpole generation, fluorescent microscopy and cryosectioning

The fragment of the *X. laevis Ras-dva1* promoter region (4026 bp) was cloned into pEGFP vector by Sal1/Age1 by Evrogen company (www.evrogen.com). The transgene frogs were generated as described previously^26^. The *in vivo* fluorescence detection was performed using the fluorescent stereomicroscope Leica M205 and photographed with Leica camera (DC 400 F). Distribution of EGFP-labeled cell were analyzed on vibrotome and cryo-sections (see Supplementary Information).

### Vivo-Morpholino injections and fin regenerating length measurements, TUNEL assay

*VivoMO*s comprise a morpholino oligonucleotide with a unique covalently linked delivery moiety, which consists of an octa-guanidine dendrimer and transferes *vivoMO* into cells where it can sequence-specifically inhibit translation of correspondent *mRNA (*www.gene-tools.com). Microinjections of the 0,4 mM solutions of sequence specific *vivo-morpholino oligonucleoides* (*vivoMO*, www.gene-tools.com), mixed with the fluorescent tracer FLD (fluorescein lysinanted dextran, Invitrogen, 40 kDa, 5 mg/ml), into the distal part of the *D. rerio* fin or *X. laevis* tail (see scheme at Supplementary Fig. S5) before and/or after the amputation as described in^8^. For *vivo-MO* sequences and their efficiency and specificity test see Supplementary Information (see Supplementary Figs S4 and S6). The measurements (square and width) of the fin regenerated area were made in the ImageJ software (http://rsb.info.nih.gov/ij). The apoptotic cells revealing assay was performed using the DeadEndTM Fluorometric TUNEL System (Promega) as described previously^8^.

### Morpholino injections and statistical analysis of malformations in regenerating tadpoles, *Ras-dva* over-expression

4–8-cell embryos were injected (4–5 nl) with the 0,25 mM water solution of correspondent *MO* mixed with the FLD as fluorescent tracer. For *MO* sequences, specificity and effectiveness tests and rescue experiment see Supplementary Information. Embryos were incubated until stage 40–42, at which their tails were inspected using fluorescent stereomicroscope Leica M205 and amputated by micro-scissors (Gills-Vannas scissors). On 7–8 dpa tadpoles with both normally and abnormally regenerated tails were counted. Statistical significance was determined with the paired sample t-test and was set P < 0,001.

To perform over-expression experiments we injected the 4–8 blastomere X. laevis embryos into the equatorial region with mRNAs of *flag-xRas-dva1* (100 ng/µl) or *xRas-dva2* (120 ng/µl) mixed with the fluorescent tracer FLD and mRNA encoding for *EGFP-xRas-dva1* (40 ng/µl)^19^. The control tadpoles were micro-injected with water solution of FLD. The expression of injected *EGFP-Ras-dva1* mRNA was detected by fluorescent microscopy imaging even 4 days after amputation (see Supplementary Fig. S9). The presence of injected *flag-xRas-dva1* and *xRas-dva2* mRNA by refractory period was detected by *in situ* hybridization (see Supplementary Fig. S9). At the stage 46 (refractory period) the tails of correspondent tadpoles were amputated and were left to regenerate for 3–5 days. In sum, 189–210 tadpoles were analyzed in three independent experiments for each mRNA variant. Statistical significance was determined with the paired sample t-test, p < 0,001

## Electronic supplementary material

Supplementary information

## Data Availability

All data generated and analysed during this study are included in this published article and in the supplementary information (Supplementary Figures S1–10).
