# Supplementary material for: Ras-dva small GTPases lost during evolution of amniotes regulate regeneration in anamniotes
Source: Sci Rep. 2018 Aug 29;8:13035. doi: 10.1038/s41598-018-30811-0 (PMC6115384; doi:10.1038/s41598-018-30811-0)
Supplement: Supplementary file 1 — Supplementary information [file 41598_2018_30811_MOESM1_ESM.pdf]

Supplementary Information

Ras-dva small GTPases lost during evolution of amniotes regulate regeneration in anamniotes.

Anastasiya S. Ivanova[1], Daria D. Korotkova[1], Galina V. Ermakova[1], Natalia Yu. Martynova[1], Andrey G. Zaraisky[1*], Maria B. Tereshina[1*]

[1] Laboratory of Molecular Bases of Embryogenesis, Shemyakin-Ovchinnikov Institute of Bioorganic Chemistry, Russian Academy of Sciences, Moscow, 117997, Russian Federation

***Corresponding authors:**
E-mail azaraisky@yahoo.com (A.G.Z.);
E-mail ma-treshka@yandex.ru (M.B.T.)

Supplementary figures and legends

Figure S1

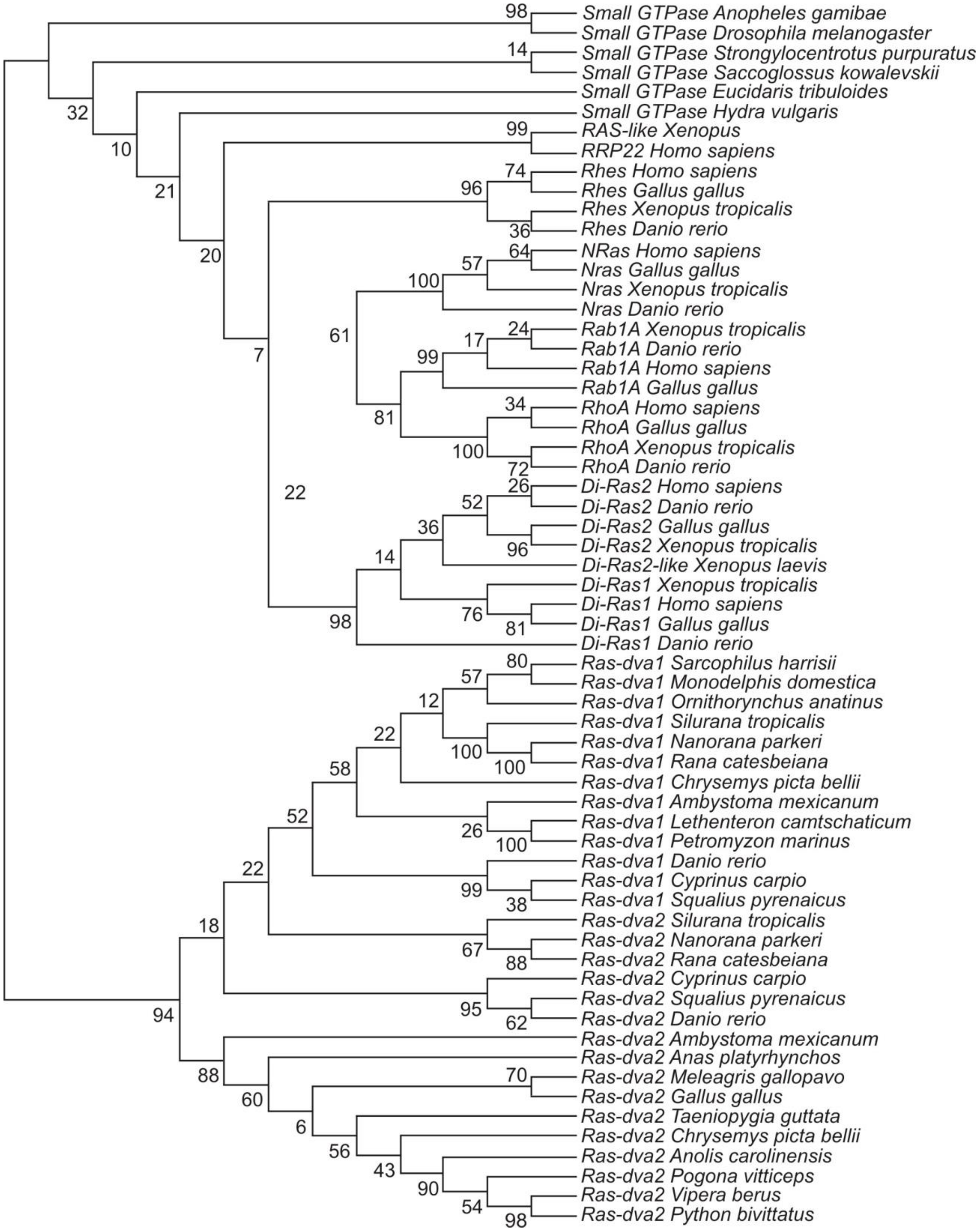

Figure S1. Phylogenetic tree of Ras-dva small GTPases and representatives of other separate groups of small GTPases.

Ras-dva small GTPases were identified in all vertebrates classes except placental mammals. These GTPases form a group (bootstrap 94) separate from Ras, Rab, Ran and other small GTPases. The subgroup of Ras-dva2 is very close to Ras-dva1 subgroup. For phylogenetic tree details and sequences numbers see Supplementary Material and Methods.

Figure S2

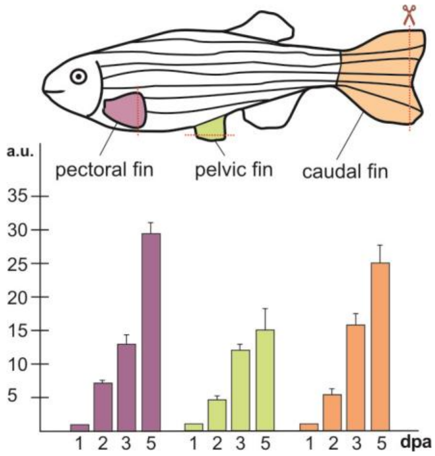

Figure S2. Outgrowth dynamics during regeneration of different *Danio rerio* fins.

To verify of regeneration score we calculated average fins height (h) outgrown by 1, 2 and 5 day post amputation (h=S/l, S- area, l - length) and compare it with each other. Drawings were done by M.B.T. The mean of the fin's h at 1dpa was used for normalization. All calculations were done with ImageJ program. For statistical analysis we carried out 5 independent experiments. Bars indicate SD. All outgrowth data at 2-5 were statistically significant, t-test, p<0,001.

Figure S3

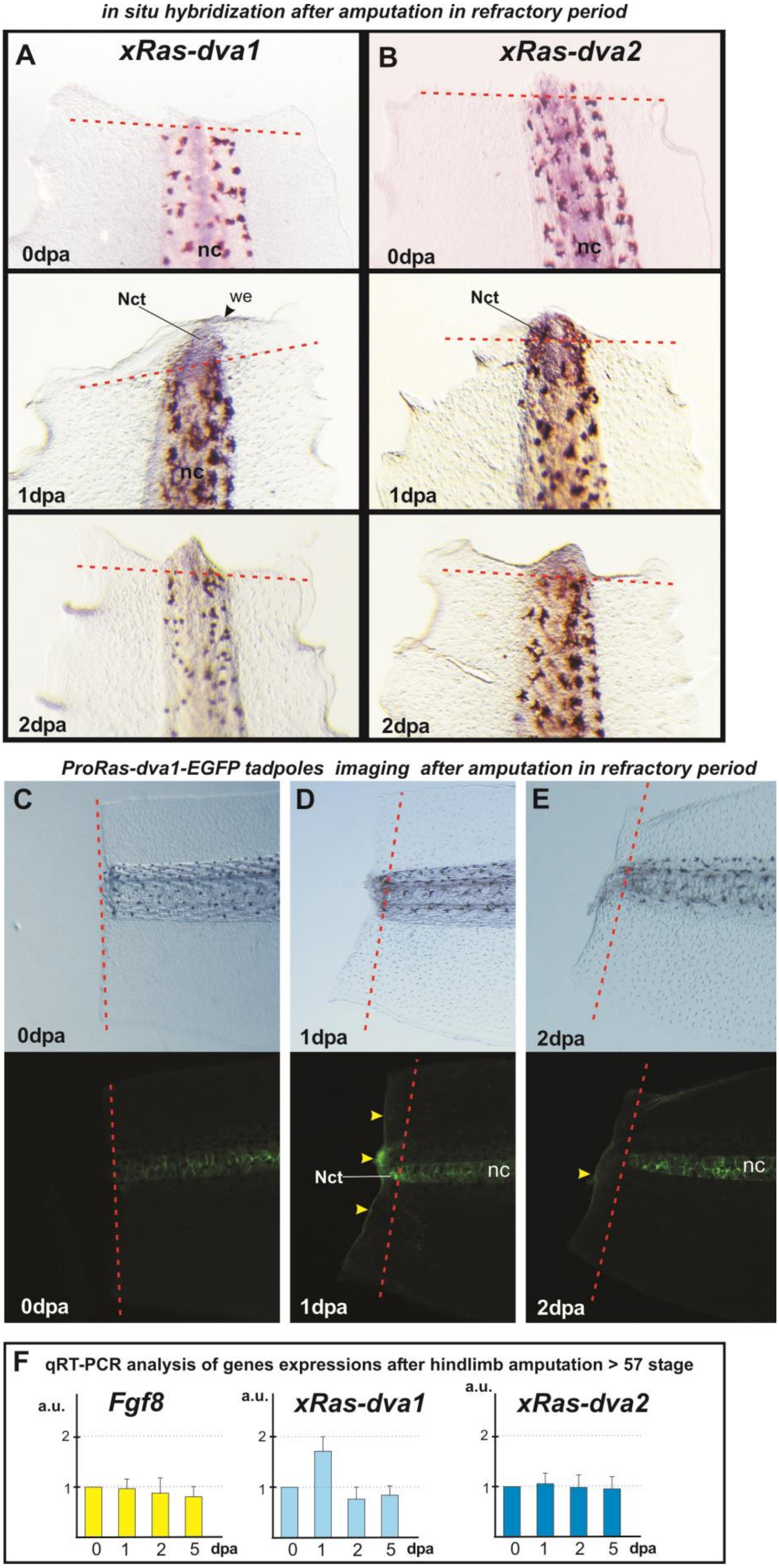
in situ hybridization after amputation in refractory period
A
xRas-dva1
B
xRas-dva2
0dpa
nc
0dpa
nc
Nct
we
Nct
nc
1dpa
1dpa
2dpa
2dpa
ProRas-dva1-EGFP tadpoles imaging after amputation in refractory period
C
D
E
0dpa
1dpa
2dpa
Nct
nc
nc
0dpa
1dpa
2dpa
F qRT-PCR analysis of genes expressions after hindlimb amputation > 57 stage
a.u.
Fgf8
a.u.
xRas-dva1
a.u.
xRas-dva2
2
1
0 1 2 5 dpa
2
1
0 1 2 5 dpa
2
1
0 1 2 5 dpa

Figure S3. Analysis of *xRas-dva* genes activity in tails and hindlimbs during non-regenerating stages.

(**A and B**) Expression pattern of *xRas-dva1 and xRas-dva2* at 0, 1 and 2 days post amputation at refractory 46 stage revealed by *in situ* hybridization. At 0dpa we detected Ras-dva1 and Ras-dva2 expression in notochord. Strong activity of gene is seen in wound epithelium and notochord tip cells at 1 dpa, but by 2 dpa expression level decreases. Nc - notochord, nct - notochord tip cells, we - wound epithelium. Dashed red line – amputation level. Dorsal to the right, distal to the top

(**C, D, E**) The *in vivo* imaging of proRas-dva1-EGFP transgenic tadpoles after amputation in refractory period. (**C**) At 0dpa we detected EGFP expression only in notochord (nc). (**D and E**) Strong *xRas-dva1* promoter activation was determined in wound epithelium (yellow arrowheads) and notochord tip cells at 1 dpa, but by 2 dpa expression level decreased and presented only in thin wound epithelium layer and notochord proximal to the amputation line.   Dorsal to the top, distal to the left. We – wound epithelium, nc – notochord, nct – notochord tip cells.

(**F**) The qRT-PCR analysis of *xRas-dva* and *Fgf8* expression dynamics in hindlimbs stumps after amputation at prometamorphic stages (57 stage and later). As can be seen only *xRas-dva1* is slightly activated at 1dpa.

Figure S4

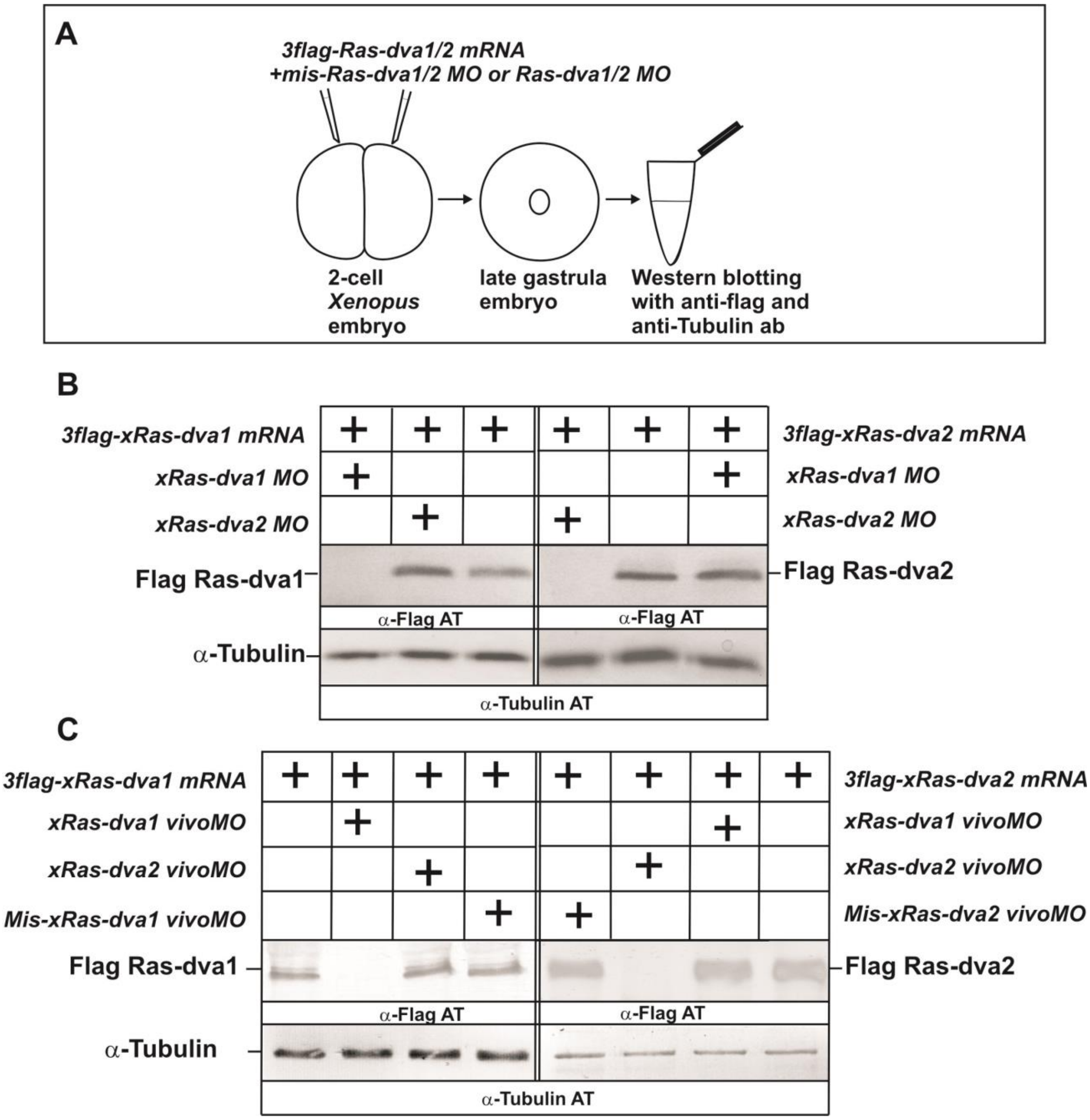

Figure S4. *Xenopus laevis* morpholino oligonucleotides (MO) and vivo-morpholino oligonucleotides (vivoMO) efficiency and specificity tests.

(A) Scheme of experiments on testing *MO/vivoMO*. mRNA encoding for *Flag-tagged xRas-dva 1* or *2* was injected into each blastomere of 2-cell *Xenopus laevis* embryos (100 pg/blastomere), either alone or with control *mis-xRas-dva1 vivoMO* (*mis-xRAs-dva2 vivoMO*) or with specific *xRas-dva1 MO/vivoMO (xRas-dva2 MO/vivoMO*) (4nl of 0,25mM *MO* solution or 4 nl of 0,4 mM water *vivoMO* solution per blastomere). The injected embryos were collected at the late gastrula stage and analyzed for presence of 3Flag-xRas-dva proteins by Western blotting with

anti-Flag antibody. Tubulin was used as loading control (see Methods). Drawings were done by M.B.T. (B) Results of western blotting with conjugated anti-flag alkaline phosphatase antibody (Sigma) and monoclonal anti-tubulin antibody demonstrate specific and effective inhibition of flag-xRas-dva1 synthesis by *xRas-dva1 MO*, but not by *xRas-dva2 MO*. The common results are obtained for *xRas-dva2 MO* demonstrating their effectiveness and specificity. (C) Results of western blotting with conjugated anti-flag alkaline phosphatase antibody (Sigma) and monoclonal anti-tubulin antibody demonstrate specific and effective inhibition of flag-xRas-dva1 synthesis by *xRas-dva1 vivoMO*, but not by *xRas-dva2 vivoMO* or *mis-xRas-dva1 vivoMO*. The common results are obtained for *xRas-dva2 vivoMO*.

Figure S5

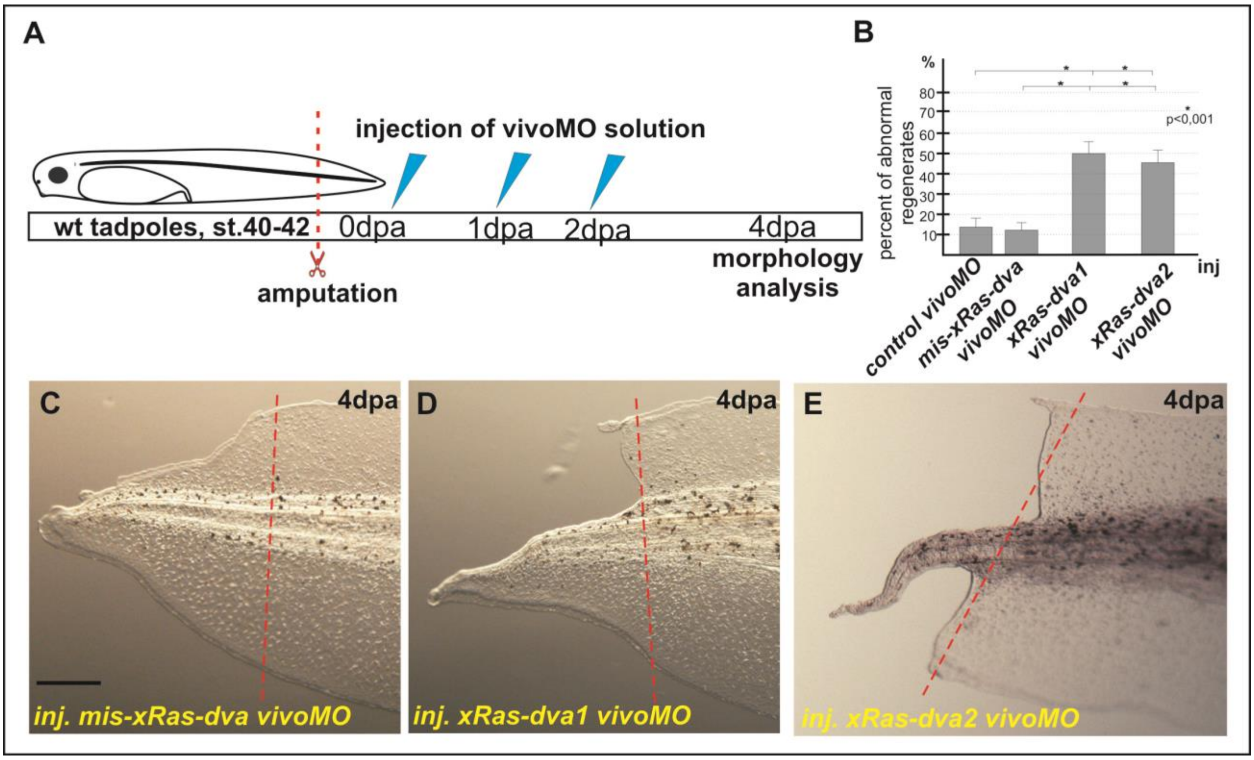

Figure S5. Down-regulation of *xRas-dva1* and *xRas-dva2* genes functioning in tail tip just after amputation results in malformations of regenerated tadpole's tails.

(A) Scheme of the experiment. The wild type tadpoles were incubated till stage 40-42. Then tadpoles' tail's distal parts were amputated and tails were injected by *vivo-Morpholino oligonucleotides* solution (*vivoMO*). *VivoMO* are able to penetrate cell membranes and can

sequence-specifically inhibit translation of correspondent mRNA. Injections were performed once per day at 0, 1, 2 dpa. At 4dpa the tadpole's regeneration success was analyzed basing on morphological parameters of restored tails. Drawings were done by M.B.T. (B) Quantification of abnormal regenerates percentage of tadpoles, injected by different *vivo-MO* solutions: *control vivoMO, mis-xRas-dva vivoMO, xRas-dva1-vivoMO and xRas-dva2-vivoMO*. Error bars indicate SD. Statistical significance of results from 5 independent experiments (1 experiment comprise 15-20 tadpoles for injection by each variant of vivoMo) was determined with paired sample t-test, the results are statistically significant, $p < 0,001$ (asterisk). (C-E) The transmitted light images of regenerated tails of 4dpa tadpoles injected by solution of *mis-xRas-dva vivoMO* (C), *xRas-dva1-vivoMO* (D) and *xRas-dva2-vivoMO* (E). The red dashed line indicates the amputation level. Scale bar 0,5 mm.

Figure S6

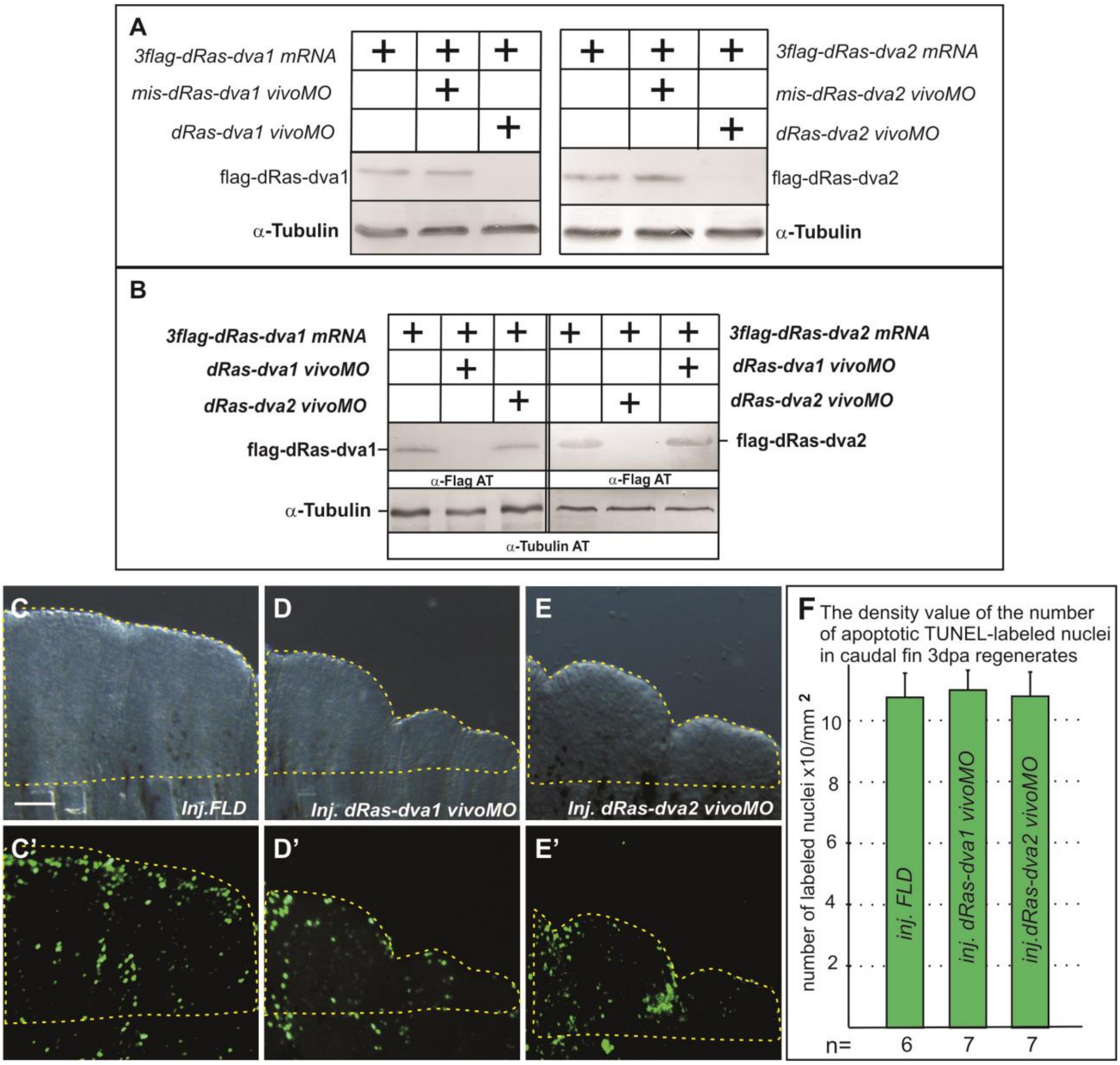

Figure S6. ***Danio Ras-dva*** **vivo-morpholino oligonucleotides efficiency and specificity test. TUNEL assay of apoptosis in the regenerating caudal fins.**

(A) Results of western blotting with conjugated anti-flag alkaline phosphatase antiboby (Sigma) and monoclonal anti-tubulin antibody demonstrate effective inhibition of flag-dRas-dva1 synthesis by *dRas-dva1vivo MO*, but not by mis-*dRas-dva1 vivoMO*. The same results are obtained for *dRas-dva2 vivoMO*. (B) *dRas-dva1 vivoMO* specifically inhibit *dRas-dva1* protein synthesis, but not *dRas-dva2* and vice versa. The scheme of the experiment was as in Supplementary Figure S4.

(C-E) Transmitted light and fluorescent images of distal areas of 3dpa caudal fins injected by fluorescent tracer (FLD) (C) or *dRas-dva1 vivoMO* and *dRas-dva2 vivoMO* (D and E). Scale bar 250µm. The bright green dots on fluorescent images indicate the apoptotic nuclei. (F) The density value of the numbers of apoptotic TUNEL-labeled nuclei in the injected fins as calculated in the regenerate's area marked by yellow dashed line. Data are represented as mean ±SD. N – number of fins used in the assay.

Figure S7

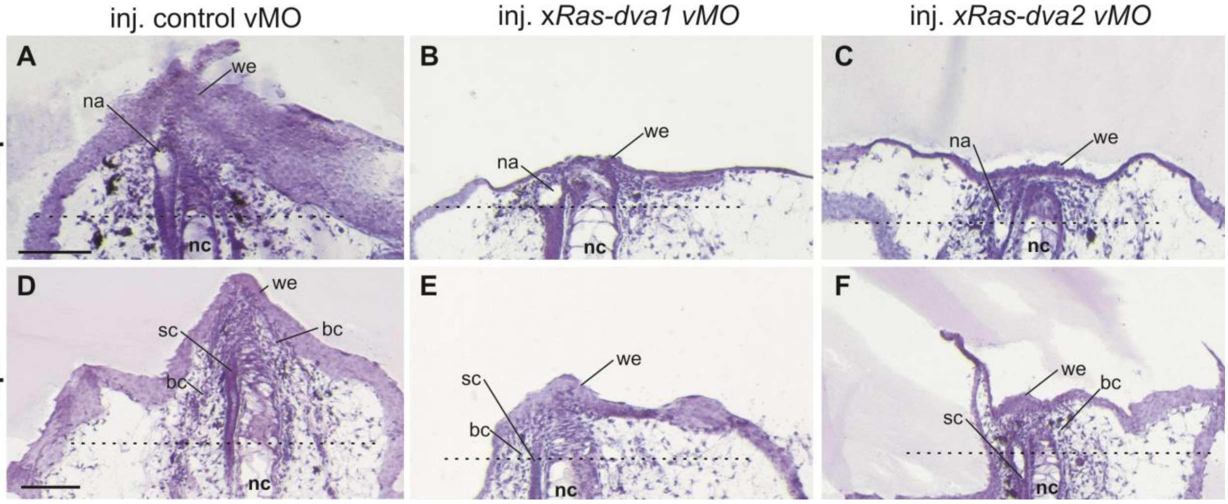

Figure S7. Histological analysis of tadpoles' tails at 1, 2 dpa upon xRas-dva1 or xRas-dva2 knock-down.

(**A-C**) Hematoxylin staining of sagittal cryosections of 1dpa tails of tadpoles injected by control vivoMO (A) or by *xRas-dva1* v*MO* (B) or *xRas-dva2 vMO* (C) tadpoles. Knock-down of Ras-dva genes results in problems in wound epithelium formation.

(**D-E**) Hematoxylin staining of sagittal cryosections of 2dpa tails of control (D) or injected by *xRas-dva1 MO* (E) or *xRas-dva2 MO* (F) tadpoles. Down-regulation of Ras-dva genes result in reduction of spinal cord and notochord regrowth and lower density of blastemal cells in contrast to control regeneration. Dashed black line indicates the amputation level. Scale bar 0,1 mm. Bc

– blastemal cells, na – neural ampule, nc – notochord, sc – spinal cord, we – wound epithelium.

Cryosections width 18µm.

Figure S8

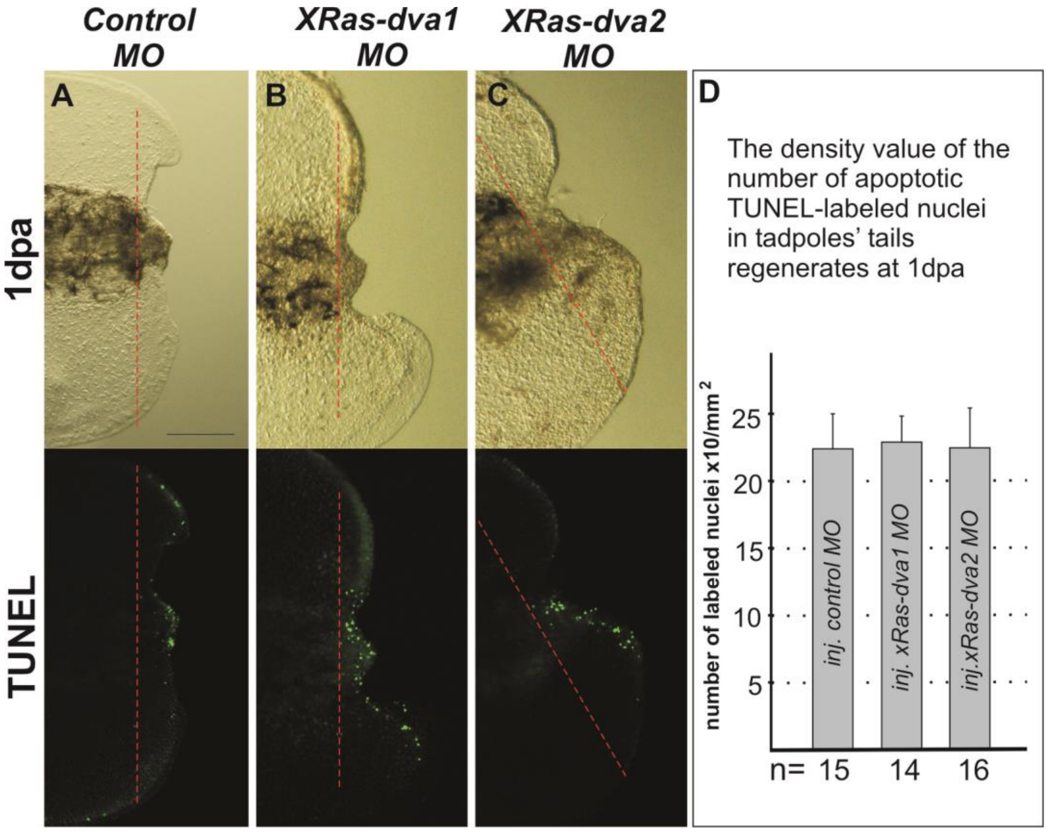

Figure S8. Apoptosis profiles in amputated tail of tadpoles with normal and inhibited Ras-dva1 or 2 functioning at 1dpa.

(A-C) Transmitted light and fluorescent images of distal areas of 1dpa tail of the tadpole, developed from embryo injected by control MO (A) or x*Ras-dva1 MO* (B) or x*Ras-dva2 MO* (C). The bright green dots on fluorescent images indicate the apoptotic nuclei after TUNEL assay. Scale bar 0,25 mm. Distal to the right, dorsal to the top. Red dashed line marks the amputation level. (D) The density value of the numbers of apoptotic TUNEL-labeled nuclei in the tadpoles tails injected by control or Ras-dva-specific MO was calculated using ImageJ software. Data are represented as mean ±SD. N – number of tails used in the assay in three independent experiments.

Figure S9

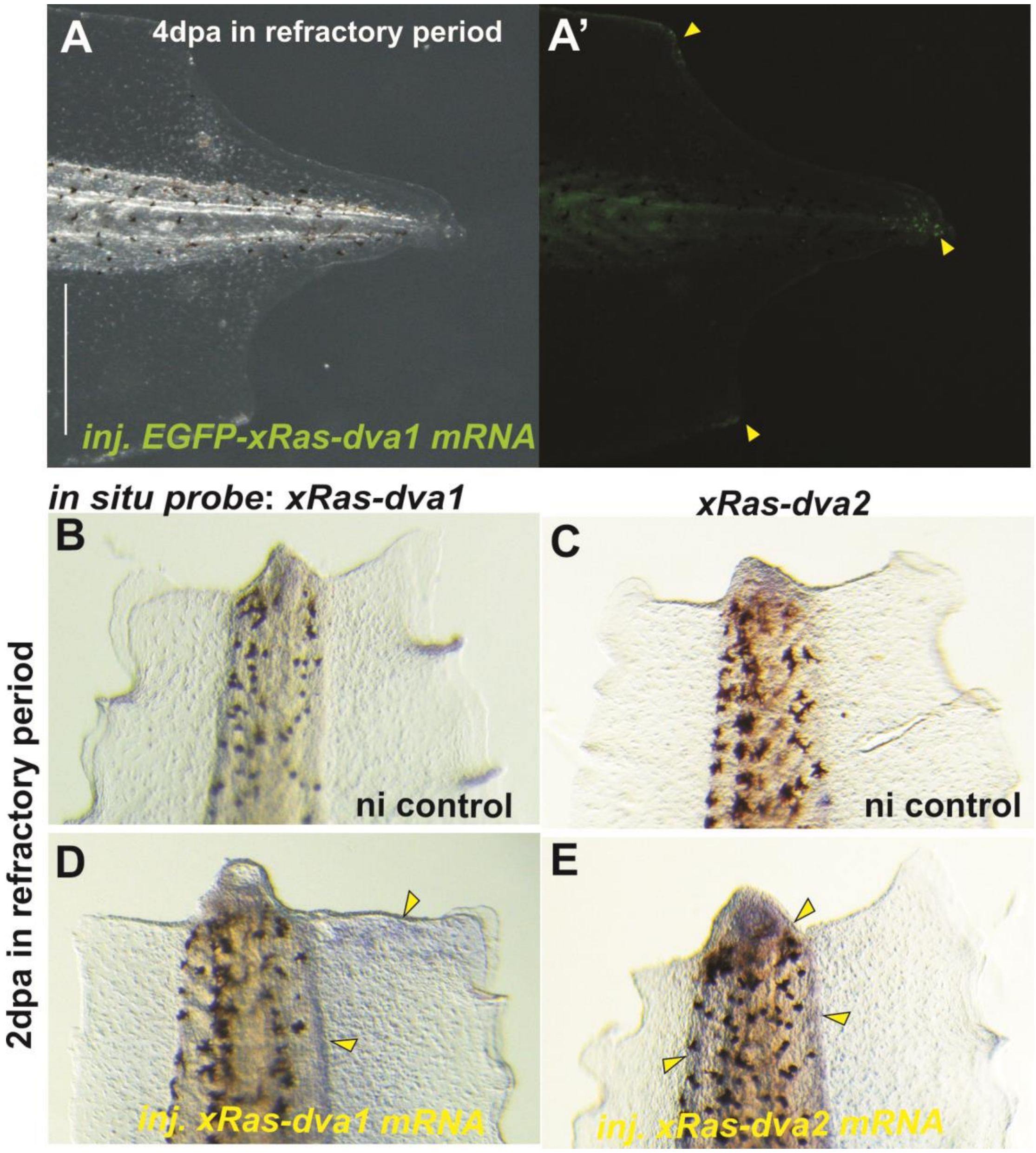

Figure S9. Detection of injected ectopic *xRas-dva1 and xRas-dva2 mRNAs* in refractory tadpoles tails after amputation.

(A and A') Transmitted light and fluorescent tail images of tadpoles developed from embryos injected by *EGFP-xRas-dva1 mRNA* and amputated in refractory period (st. 46). Fluorescent signals in regenerating refractory tail in fins and tip demonstrate that injected mRNA is still functions. Scale bar 2mm. (B and C) *In situ* hybridization staining of control refractory tadpole's

tails at 2dpa for *xRas-dva1* and *xRas-dva2* expression show weak signal in the tail tip. (D and E) *In situ* hybridization staining of refractory tadpole's tails developed from embryos, which were injected at 2-4 cell stage by synthetic *xRas-dva1* or *xRas-dva2 mRNA*, demonstrate *xRas-dva1* (D) or *xRas-dva2* (E) ectopic *mRNA* presence in tail tip, fins and muscles (yellow arrowheads) at 2dpa.

Figure S10.

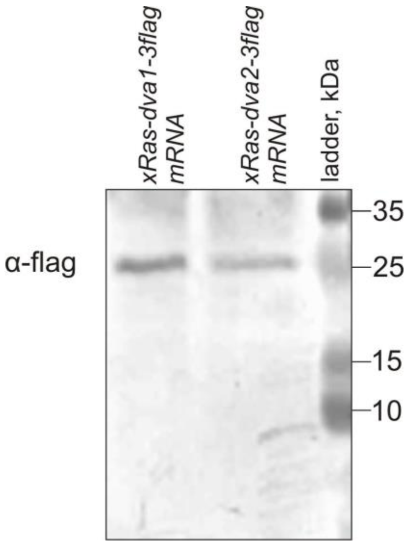

Figure S10. Synthetic mRNA viability test.

Results of western blotting with conjugated anti-flag alkaline phosphatase antiboby (Sigma) demonstrate translation activity of synthetic mRNA *xRas-dva1-3flag* and *xRas-dva2-3flag* in samples of refractory tail's tips at 2dpa of tadpoles, developed from embryos injected by correspondent mRNAs.

Supplementary materials and methods

Phylogenetic analysis.

All small GTPases contain G-domain which is necessary for its GTPase activity and specific effectors binding. The flanking N- and C-ends differ a lot in different small GTPases and make

alignment of their whole aa-sequences a trickish problem for most Clustal versions. Thus for the phylogeny analysis we used alignment of G-domains of different Ras-like small GTPases from subfamilies Ras-dva, Rhes, Rab, NRas, Di-Ras and Rho which is sufficient for proper clustering of proteins in separate known groups (families). The phylogeny analysis was made by MEGA 6.06 software. As result we constructed maximum likehood tree using bootstrap method (number of bootstrap replications 500) and model of Jones-Taylor-Thornton (JTT) (Supplementary Fig. S1).

Below are aa-sequences (for Ambystoma mexicanum Ras-dva), GeneBank accession numbers and web address (for Ras-dva1 Ornithorynchus anatinus) of Ras-dva sequences in species presented on phylogenetic tree in Supplementary Figure S1:

>Ras-dva1 (G-domain) Ambystoma mexicanum

(https://www.ncbi.nlm.nih.gov/bioproject/PRJNA300706)

GAAGVGKTALIQRFLTGRFESRHRRTVEELHSLDCELGPRRVSLEVLDTSGSYAFPAMR

KLSIRRAQAVALVFSLAEPGSFEEVQRLREEVAELRADEDPPLPVMVVGNQADLFPGGL

LCRPPLAEHLAATAELEWGCTYLETSAK

>Ras-dva2 (G-domain) Ambystoma mexicanum

GAAGVGKTALIRRFLLDTFETKYKRTVEELHTKEYEVSGMTFTIEIMDTSGSYSFPAMRK

LSIQNSDAFALVYAIDDAESFQSVKSLRDEILETKEDKMAPIVVVGNKVDSEEGRQVATE

ETLSLVELEWNNRFVEASAK

>Ras-dva1 Silurana tropicalis (AAU45400)

>Ras-dva2 Silurana tropicalis (NP_001037874)

>Ras-dva1 Rana catesbeiana(LIAG010363413)

>Ras-dva2 Rana catesbeiana (LIAG010304562)

>Ras-dva1 Nanorana parkeri (JYOU01005177)

>Ras-dva1 Nanorana parkeri (JYOU01027940)

>Ras-dva1 Danio rerio (NP_001073403)

>Ras-dva2 Danio rerio (AAU45399)

>Ras-dva1 Squalius pyrenaicus (CVRK01004533)

>Ras-dva2 Squalius pyrenaicus (CVRK01012534)

>Ras-dva1 Cyprinus carpio (LHQP01052524)

>Ras-dva2 Cyprinus carpio (LHQP01001664)

>Ras-dva1 Chrysemys picta bellii (AHGY01055857)

>Ras-dva2 Chrysemys picta bellii (AHGY01500890)

>Ras-dva2 Pogona vitticeps (CEMB01025440)

>Ras-dva2 Anolis carolinensis (AAWZ02009177)

>Ras-dva2 Python bivittatus (AEQU02041064)

>Ras-dva2 Vipera berus (JTGP01070677)

>Ras-dva2 Gallus gallus (AAU45401)

>Ras-dva2 Meleagris gallopavo (ADDD02006242)

>Ras-dva2 Anas platyrhynchos (ADON01074023)

>Ras-dva2 Taeniopygia guttata (ABQF01054136)

>Ras-dva1 Monodelphis domestica AAFR03028671

>Ras-dva1 Ornithorynchus anatinus

(http://www.ncbi.nlm.nih.gov/Traces/trace.cgi?cmd=retrieve&val=605033602)

>Ras-dva1 Petromyzon marinus (ABY86653)

>Ras-dva1 Lethenteron camtschaticum (APJL01043175)

>Ras-dva1 Sarcophilus harrisii (AFEY01443738)

>RRP22 Homo sapiens (NM_006477)

>small GTPase Saccoglossus kowalevskii (XP_002732228)

>small GTPases Eucidaris tribuloides (JZLH010803589)

>small GTPases Strongylocentrotus purpuratus (XP_011662785.1)

>small GTPases Hydra vulgaris (ACZU01012823.1)

>small GTPases Drosophila melanogaster (AAF45493)

>small GTPase Anopheles gambiae (XM_309343)

>RAS-like Xenopus (NP_001090589)

>Di-Ras2-like Xenopus laevis (XP_018113692)

>Di-Ras1 Xenopus tropicalis (NP_001072780)

>Di-Ras2 Xenopus tropicalis (XP_002937072.1)

>Di-Ras1 Danio rerio(NP_001119893.1)

>Di-Ras2 Danio rerio (XP_005155609.1)

>Di-Ras1 Gallus gallus(XP_015155454.1)

>Di-Ras2 Gallus gallus (XP_004949308.1)

>Di-Ras1 Homo sapiens (NP_660156.1)

>Di-Ras2 Homo sapiens (NP_060064.2)

>RhoA Danio rerio (NP_997914.2)

>RhoA Xenopus tropicalis (XP_012819968.1)

>RhoA Gallus gallus(CAC08447.1)

>RhoA Homo sapiens (NP_001300870.1)

>Rab1A Danio rerio (AAH62857.1)

>Rab1A Xenopus tropicalis(AAI27357.1)

>Rab1A Gallus gallus (NP_001257591.1)

>Rab1A Homo sapiens (CAG38727.1)

>Rhes Danio rerio(NP_001025373)

>Rhes Xenopus tropicalis (NP_001016006.1)

>Rhes Gallus gallus (XP_416293.3)

>Rhes Homo sapiens (NP_055125.2)

>NRas Danio rerio (NP_571220.2)

>NRas Xenopus tropicalis (NP_001016763.1)

>NRas Gallus gallus (NP_001012567.1)

>NRas Homo sapiens (NP_002515.1)

Cloning of *Danio rerio Ras-dva1* and *Ras-dva2* cDNA fragments. Cloning flag-tagged Ras-dva 1/2. Dig-probe synthesis.

To obtain the template for synthesis of the d*Ras-dva1* and d*Ras-dva2* dig-probes, DNA fragment corresponding to the protein coding regions of *dRas-dva1* and *dRas-dva2* cDNAs were obtained by RT-PCR from the first strand of the *Danio rerio* embryos (60 hpf) total RNA with the following primers:

dRas-dva1 forward 5'-AATGAATTCGGGAAGATGTCTCTGTTAGTGCAG

dRas-dva1 reverse 5'- CTTAAGCATCAGTATGATGCATAACTAC

dRas-dva2 forward 5'- AATGAATTCGCCACCATGTCTCTGGAAGTCAAG

dRas-dva2 reverse 5'- CTTCAAGGATCCGTCCACATCA

The amplified PCR cDNA fragments of *dRas-dva1* and *dRas-dva2* were cloned to pGEM-T vector (Promega) and correct clones were selected by virtue of sequencing of three random clones. The Dig-labeled RNA antisense probe for the whole-mount *in situ* hybridization was synthesized by T7 or SP6 polymerase (mMessageMachine) from the PCR-product obtained with *dRas-dva1* forward and *M13* forward or *dRas-dva2* reverse and *M13* reverse primers from *dRas-dva1pGEM-T* or *dRAs-dva2pGEM-T* plasmid constructs respectively. The control sense dig-RNA probes were synthesized by SP6 or T7 polymerases from the PCR-products obtained with *dRas-dva1* reverse and M13 reverse or *dRAs-dva2* forward and M13 forward primer pairs.

To construct templates for testing the efficiency of *Danio* and *Xenopus Ras-dva1* and *Ras-dva2 VivoMO*, the respective cDNAs were obtained by PCR with the following primers (restriction sites of EcoRV and NcoI are underlined):

dRas-dva1 forward 5' - AATGATATCGGGAAGATGTCTCTGTTAGTGCAG

dRas-dva1 reverse 5' – CGGGCCCCATGGTTAAGACAAGATGCAGCTG

dRas-dva2 forward 5'-TTGATATCCGTCAAGGAACCATGTCTCTGGAAG

dRas-dva2 reverse 5' - CGGGCCCCATGGTTATGAGACGGAGCAGCTGTTAGT

xRas-dva1 forward 5' - AATGATATCGGCACCATGTCTGTATCCTCCA

xRas-dva1 reverse 5' – CGGGCCCATGGCTTTAGGATAAAGTG

xRas-dva2 forward 5' - AATGATATCGGAGCC**ATG**TCACTGTCCACAA

xRas-dva2 reverse 5' – CGGGCCCCATGGAGATCAGCCGATGCT

The obtained cDNAs were sub-cloned into *pCS4-3flag* by EcoRV and NcoI, upstream and in frame with the last 3flag-tags. Capped synthetic mRNAs encoding for *Danio* or *Xenopus Ras-dva1-3flag* and *Ras-dva2-3flag* were synthesized with SP6 Message Machine Kit (Ambion) using the obtained plasmids cut by NotI.

mRNA viability test.

To check viability of exogenous mRNA at refractory period stages we injected xRas-dva1-3flag and xRas-dva2-3flag mRNA (100pg/blastomere, cloning and RNA synthesis of which are described in above) into 8-16 blastomere stage embryos in equatorial cell to guide mRNA to cells precursors of the tail bud. Further at stage 46 amputated tails in refractory period, incubated tadpoles till 2dpa and fixed tail tips (40-50 pieces per sample) for western blotting analysis with conjugated anti-flag alkaline phosphatase antiboby (Sigma). As a result we detected presence of Ras-dva1-3flag and Ras-dva2-flag proteins in refractory tails at 2dpa (see Fig.S10). In our mind, this data clearly demonstrate that injected synthetic mRNA is viable at least till stage 46 and justifies overexpression experiments during refractory period.

qRT-PCR primers

The following pairs of primers for *Danio rerio* (d) and *Xenopus laevis* (x) genes were used for qRT-PCR:

dRas-dva1 forward 5'-CAACAGATGAAGTTACCGA;

dRas-dva1 reverse 5'-ACATTTTCATATAAATGTCC;

dRas-dva2 forward 5'-AAGGTGGAGATGGACTGGAAC;

dRas-dva2 reverse 5'-GCCGTAGACTCAAGTCTTTAG;

xRas-dva1 forward 5'- TACCGGCGCACAGTGGAGGA

xRas-dva1 reverse 5'- GGCACCTCGGCGTCTCCTTT

xRas-dva2 forward 5'- GGCGACCTGAAGGTGTCTTG

xRas-dva2 reverse 5'- TCCTCCTCAGTGCCGGGCTT

xFgf20a forward 5'- ATCACAGCCGATTCGGTATCC

xFgf20a reverse 5'- CTCAAACTGTTCCCGAAAAATGC

xMsx1b forward 5'- TCTCCTATGGGACTTTACACA;

xMsx1b reverse 5'- AATCACTCAAGTCTTCTTTC;

xFgf8 forward 5'- CTGCGTCTTCTCGGAAATTGTC;

xFgf8 reverse 5'- TTTGGCAACCTCTTCATGAAGT;

Primers for the *X. laevis xAg2, ODC* and *EF-1alpha* and *D. rerio ODC*, *EF-1alpha, Fgf20a, dAg1, dAgr2* and *Igf2b* were the same as in [1,2].

PCR efficiency (PE) for *xRas-dva1, xRas-dva2, dRas-dva1, dRas-dva2* was calculated as described previously and were respectively the following: 1,801, 1,793, 1,799, 1,808.

The geometric mean of expression of two reference housekeeping genes: *ornithine decarboxylase (ODC)* and *elongation factor 1alpa (EF-1alpha)* was used for normalization of the target genes expression levels. The qRT-PCR data for each gene expression was obtained and calculated using total RNA derived from three-five independent samples. The value of normalized PCR signal of the 0dpa sample, harvested immediately after amputation, was taken as an arbitrary unit (a.u.) in each series.

***Morpholino oligonucleotides (MO)* sequences and efficiency/specificity tests.**

The main tool for reverse genetics in *Xenopus* and *Danio* species have become anti-sense morpholino oligonucleotides (MO) injections. In contrast to RNAi, which appeared to be inefficient or non-specific in *Xenopus* and *Danio*, anti-sense MO were proved to be more efficient than conventional *DNA* or *RNA* oligonucleotides. Following initial injections into frog

or fish embryos at the single-cell or few-cell stages, MO effects can often be measured five days later, after most of the processes of organogenesis and differentiation are past, with observed phenotypes consistent with target-gene knockdown [3]. The precise MO sequence design and proper controls with mis-targeted MO make this tool for modification of target gene activity, firstly, very effective and, secondly, makes possible rapid achievement of results of phenotypic consequences analysis. In our work we used the following antisense morpholino oligonucleotides (MO) (Gene Tools) to block the translation of *xRas-dva1* and *xRas-dva2* endogenous mRNAs:

xRas-dva1 MO 5'- GTGAGATTGCGCTTTCTTTTGTCTG

xRas-dva2 MO 5'-TCTTCTCCTTTGTGGACAGTGACAT

Microinjections of commercial *control MO* provided by Gene Tools and *mis-Ras-dva1* and *2 MO* with mismatches (underlined) were used as controls:

mis-xRas-dva1 MO 5'-GTGACATTGCTCTTTCTTTTGTGTT

mis-xRas-dva2 MO 5'-TCATCTGCTTTATGGACATTGACAG

To test efficiency and specificity of MO *in vitro* we injected mRNA encoding for *3Flag-tagged xRas-dva 1* or *2* into each blastomere of 2-cell *Xenopus laevis* embryos (100 pg/blastomere), either alone or with *xRas-dva1 MO* or with *xRas-dva2 MO* (4 nl of 0,25 mM water solution/blastomere). The injected embryos were collected at the late gastrula stage and analyzed for presence of 3Flag-xRas-dva proteins by Western blotting with Sigma Anti-flag antibody (cat. # A9469). Tubuline was used as loading control (see the scheme of experiment in Supplementary Fig. S4). The results obtained confirm the efficiency and specificity of *xRas-dva1/2 MO* used.

Besides to check specificity of MO used *in vivo* we performed *xRas-dva1 MO* rescue experiment. We used mRNA, encoding for *flag-xRas-dva1* missing the morpholino target sequence located in the 3'UTR region of *xRas-dva1* gene, which was cloned previously [4]. The embryos were injected either with *xRas-dva1 MO* (0,25mM) or with *xRas-dva1 MO*

(0,25mM) mixed with *flag-xRas-dva1 mRNA* (100ng/mkl). To test the specificity of *xRas-dva2 MO* effects we obtained *xRas-dva2* mRNA missing the morpholino-binding site. To this we used the following forward primer (mismatches to MO site are underlined) 5’-AAGAATTCACTAGCCATGAGTTTATCAACAAAAGAA. The PCR-product was subcloned into pCS2 plasmid construct and transcribed by the SP6-polymerase (mMessage Machine Kit (Ambion)). The co-injection of *flag-Ras-dva1* and *xRas-dva1 MO* or *mismatched xRas-dva2 mRNA*(40ng/mkl) with *xRas-dva2 MO* rescued the regeneration abnormalities and represented 73% and 78% of normally regenerating tadpoles out of 150 and 165 analyzed in three independent experiments (see Fig. 5F).

***Vivo-Morpholino Oligonucleotides (vivoMO)* sequences and efficiency/specificity tests.**

The following vivo-MOs were used:

dRas-dva1 vivoMO 5’ ACAGAGACATCTTCCCTCAGTTATT

dRas-dva2 vivoMO 5’ CTTCCAGAGACATGGTTCCTTGACG

xRas-dva1 vivoMO 5’ CAGTGCTTTACTCCAGAGGCAGGAG

xRas-dva2 vivoMO 5’ TCTTCTCCTTTGTGGACAGTGACAT

As the control, we used microinjections of commercial *control vivoMO* provided by Gene Tools and *mismatched Ras-dva vivoMO* with four-five mismatches (underlined):

control VivoMO 5’ TCTGTGGATGTCTTGCTCTTCCAGG;

mis-dRas-dva1 vivoMO 5’ ACAGACACGTCATCCGTCAGGTATT

mis-dRas-dva2 vivoMO 5’ CTTCAAGTGACGTGGTACCTTTACG

mis-xRas-dva1 vivoMO 5’ CAGTCCTTAACTGCAGACGCACGAG

mis-xRas-dva2 vivoMO 5’ TCATCTGCTTTCTGGACACTGACAT

In the down-regulation experiments as control we used mix of mis-xRas-dva1 and mis-xRas-dva2 vivoMO which was named mis-xRas-dva vivoMO (see Supplementary Fig. S5).

To test efficiency and specificity of *Danio* and *Xenopus Ras-dva1* and *Ras-dva2 vivoMO,* we injected *Xenopus* embryos with either mRNAs encoding for flag-tagged *Ras-dva1/ Ras-dva2* alone (100pg/blastomere) or together with *Ras-dva1 vivoMO* (0,4mM), *mis-Ras-dva1 vivoMO* , *Ras-dva2 vivoMO* (0,4mM) or *mis-Ras-dva2 vivoMO,* respectively (see scheme of experiment in Supplementary Fig. S4). The injected embryos were collected at the blastula-early gastrula stage and analyzed for the presence of Danio or Xenopus Ras-dva1-3flag and Ras-dva2-3flag proteins by Western blotting with Sigma Anti-flag antibody (cat. # A9469) as described previously [5]. Tubulin was detected by monoclonal anti-tubulin antibody, obtained in the Institute of protein RAS, and was used as loading control.

Strong suppression of *Ras-dva1-3flag* and *Ras-dva2-3flag mRNAs* translation was observed in embryos microinjected with these mRNAs and the respective *Ras-dva1 vivoMO* and *Ras-dva2* v*ivoMO* (Supplementary Fig. S4 and S6). In contrast, co-injection of *mismatched vivoMO* (*misRa*s*-dva1* or *misRas-dva2*) didn't show any *Ras-dva1* or *Ras-dva2* mRNA translation inhibition. These results confirm the efficiency of *dRas-dva1, dRas-dva2, xRas-dva1* and *xRas-dva2 vivoMO*.

To test the specificity of these *vivoMO* we tested the effects of *vivoMO* on translation of the most relative small GTPases, namely*, dRas-dva1 vivoMO* on dRas-dva2 protein and *dRas-dva2 vivoMO* on dRas-dva1. For this we co-injected *3flag-dRas-dva1 mRNA* with *dRas-dva1 vivoMO* or *dRas-dva2 vivoMO* and *3flag-dRas-dva2 mRNA* with *dRas-dva2 vivoMO* or *dRas-dva1vivoMO*. The following analysis was performed according to the described in Figure S4 scheme. Specific inhibition of dRas-dva1 translation by *dRas-dva1 vivoMO* but not *dRas-dva2 vivoMO* was detected, and vice versa (Supplementary Fig. S6). These results confirm the specificity of *dRas-dva1/2 vivoMO* used. All the same tests were made for *xRas-dva1 vivoMO* and *xRas-dva2 vivoMO* (Supplementary Fig. S4). The results obtained confirm the efficiency and specificity of *Xenopus* and *Danio Ras-dva1/2 vivoMO* used.

Vibratome- and cryo- sections.

After fixation in 4%PFA the *Xenopus* tails samples were transferred into 5% agarose (3:1 agarose: low melt agarose), where the tip of the sample was oriented and let harden overnight in +4C. Further vibratome sectioning (40µ thick) was held in accordance with the manual guideline (Microm HM 650 V). The *Xenopus* tail, hindlimb buds and *Danio* caudal fins (the cryosectioning samples) after fixation in 4% PFA were transferred to the melted warm (+47°C) 1,5% bacto-agar on 5% sucrose solution and were oriented in it till the sample curdled. The cube with the sample was left in 30% sucrose solution for 12-15 hours and then was bound by the Neg-50 (Richard-Allan Scientific) to the specimen holder and covered also by Neg-50. Further, the holder with the sample was carefully inserted into a liquid nitrogen and then cryosectioned (7-20µ thick) on the Microm HM 525 (Thermo Scientific) and placed on superfrost plus microscope slides (Fisher Scientific, cat.# 12-550-15).

The sections were then used for hematoxylin staining or *in situ* hybridization according the protocol, described by Liu and colleagues [6].

Immunohistochemistry staining.

The samples of *Xenopus* tails were fixed in 4% PFA solution overnight and then washed by the following solutions: 1xPBS solution (3 washes, 5-10 minutes each), PBT (1xPBS, 0,2% tween 20) (3 washes, 10 minutes each) and with blocking solution (10% newborn calf serum (Sigma cat. # N4762) in PBT) (60 minutes each) and then incubated with primary anti-bodies diluted in the blocking solution overnight at +4C.

The following antibodies were used:

for cell proliferation staining - rabbit anti- phospho-Histone H3 (Ser10) mitosis marker (pH3) (Millipore, cat. # DAM1545035, 1:100). The primary antibodies were washed by 3 washes with PBT followed by blocking solution and incubation with secondary antibodies: anti-rabbit or anti-mouse IgG (H+L) fragments conjugated with fluorescent CF568 protein (Sigma, cat. #

SAB4600400 and #SAB4600425 respectively, 1:500). After overnight incubation we performed 3 PBS washes and imaging using fluorescent stereomicroscope Leica M205. The number of fluorescent cells were counted in ImageJ. For statistical analysis we used two-tailed t-test, $p<0,05$.

References

1. Ivanova, A. S. *et al.* The secreted factor Ag1 missing in higher vertebrates regulates fins regeneration in Danio rerio. *Sci. Rep.* **5,** 8123 (2015).
2. Ivanova, A. S., Tereshina, M. B., Ermakova, G. V, Belousov, V. V & Zaraisky, A. G. Agr genes, missing in amniotes, are involved in the body appendages regeneration in frog tadpoles. *Sci. Rep.* **3,** 1279 (2013).
3. Hardy, S., Legagneux, V., Audic, Y. & Paillard, L. Reverse genetics in eukaryotes. *Biol. cell* **102,** 561–80 (2010).
4. Tereshina, M. B., Ermakova, G. V, Ivanova, A. S. & Zaraisky, A. G. Ras-dva1 small GTPase regulates telencephalon development in Xenopus laevis embryos by controlling Fgf8 and Agr signaling at the anterior border of the neural plate. *Biol. Open* **3**, 192-203, (2014).
5. Eroshkin, F. M., Fedina, N. V, Martynova, N. Y., Bayramov, A. V & Zaraisky, A. G. [The Point Mutation in NOGGIN2 Protein That Enhances Its Ability to Bind Activin]. *Bioorg. Khim.* **41,** 749–751 (2015).
6. Liu, K., Wang, X., Li, Z., He, R. & Liu, Y. in *Methods in molecular biology (Clifton, N.J.)* **1082,** 129–141 (2014).
